# Computational and Statistical Analyses of Amino Acid Usage and Physico-Chemical Properties of the Twelve Late Embryogenesis Abundant Protein Classes

**DOI:** 10.1371/journal.pone.0036968

**Published:** 2012-05-16

**Authors:** Emmanuel Jaspard, David Macherel, Gilles Hunault

**Affiliations:** 1 Université d'Angers, Institut de Recherche en Horticulture et Semences (INRA, Agrocampus-Ouest, Université d'Angers), SFR 4207 QUASAV, LUNAM Université, Angers, France; 2 Université d'Angers, Laboratoire d'Hémodynamique, Interaction Fibrose et Invasivité tumorale hépatique, UPRES 3859, IFR 132, LUNAM Université, Angers, France; University of South Florida College of Medicine, United States of America

## Abstract

Late Embryogenesis Abundant Proteins (LEAPs) are ubiquitous proteins expected to play major roles in desiccation tolerance. Little is known about their structure - function relationships because of the scarcity of 3-D structures for LEAPs. The previous building of LEAPdb, a database dedicated to LEAPs from plants and other organisms, led to the classification of 710 LEAPs into 12 non-overlapping classes with distinct properties. Using this resource, numerous physico-chemical properties of LEAPs and amino acid usage by LEAPs have been computed and statistically analyzed, revealing distinctive features for each class. This unprecedented analysis allowed a rigorous characterization of the 12 LEAP classes, which differed also in multiple structural and physico-chemical features. Although most LEAPs can be predicted as intrinsically disordered proteins, the analysis indicates that LEAP class 7 (PF03168) and probably LEAP class 11 (PF04927) are natively folded proteins. This study thus provides a detailed description of the structural properties of this protein family opening the path toward further LEAP structure - function analysis. Finally, since each LEAP class can be clearly characterized by a unique set of physico-chemical properties, this will allow development of software to predict proteins as LEAPs.

## Introduction

«Late Embryogenesis Abundant» proteins (LEAPs) were originally discovered in cottonseeds thirty years ago as hydrophilic polypeptides whose transcripts were massively synthesized during late seed maturation [1]–[5]. They are especially prominent in plants with up to 71 genes annotated as LEAPs in *Arabidopsis* [6]–[8]. LEAPs have been identified also in bacteria, fungi, algae and animals [9]–[12] and are associated with abiotic stress tolerance, particularly dehydration, cold stress and salt stress [3], [13]–[15], suggesting a general protective role in anhydrobiotic organisms. However, in spite of their abundance and expected major role in desiccation tolerance, their structural features and molecular functions still remain largely unknown.

LEAPs are highly hydrophilic proteins with repeated amino acid motifs, and peculiar structural features since they are generally unstructured polypeptides with a propensity for alpha-helix formation [16]. This is well illustrated by the case of LEAM, a LEAP from pea seed mitochondria [17], which, in the hydrated state behaved as an intrinsically disordered polypeptide localized in the matrix space. However, upon dehydration, LEAM was shown to fold into a helical form that was able to immerse laterally within the inner layer of the inner membrane, reinforcing the membrane in the dry state [17]–[19]. This insertion mechanism is fully reversible upon imbibition, when LEAM unfolds and leaves the inner membrane, avoiding interference with the energy transducing membrane in the hydrated state [18].

Despite such a role in membrane protection, and some theoretical studies such as molecular dynamics simulations [10], the functional mechanism of most LEAPs at the molecular level remains to be demonstrated (*i.e.*, no clear partner or cellular target has been yet identified). Investigating the structure - function relationships of LEAPs is thus of primary interest, but remains challenging because experimental evidence is difficult to obtain, especially when considering biochemical and biophysical analyses in the dry state. Since many LEAP sequences are now available and have been gathered into a dedicated database (LEAPdb [8]), computational analyses of the amino acid sequences offer an alternative approach to get novel insights into the molecular characterization and function of LEAPs. LEAPdb contains 710 LEAP sequences, and the whole set has been organized in 12 non-overlapping classes corresponding to 8 PFAM (PF00257, PF00477, PF02987, PF03168, PF03242, PF03760, PF04927, PF10714).

As pointed out above, most LEAPs are expected to lack defined structure in the hydrated state, which classifies them as natively unfolded or intrinsically disordered proteins (IDPs). IDPs and disordered regions in proteins challenge the structure -function dogma because they are final products of protein biogenesis contributing to cellular functions without a well defined three-dimensional structure [21]–[23], at least until they interact with their cellular partner. In agreement with their lack of defined structure in the native state, LEAPs are seldom represented in protein structure databases. There are only two available 3-D structures corresponding to *Arabidopsis* LEAPs: PDB code 1XO8 coded by At1g01470 [24] and PDB code 1YYC coded by At2g46140. Both are members of the PFAM family PF03168. Although the classification of these two proteins as LEAPs was previously debated [25], it is likely that they can be considered as genuine LEAPs [7]–[8]. Within the LEAPs family essentially composed of IDPs, there is thus at least one fully natively folded sub-family, which strengthens the interest toward mining the protein sequences features on a large scale. For that purpose, we have used as a resource the LEAP sequences originally deposited in LEAPdb.

A large number of physico-chemical properties and amino acids usage of the 12 LEAP classes have been computed and statistically analyzed. Although LEAPs are generally known to be IDPs, we provide evidence that LEAPs from class 7 (PF03168), and probably those of class 11 (PF04927), are natively folded. Although LEAP classification has been often updated [5], [14], [25]–[27], no clear rule has yet emerged to classify these proteins unambiguously. Here, we provide a validation and an exhaustive characterization of the 12 LEAP classes previously described [8], based on robust computational and statistical analyses of amino acids physico-chemical properties. This will aid an understanding of the evolution, structure and function of these enigmatic proteins. Moreover, clear characterization of each LEAP class by a unique set of properties, will help with the development of software to predict proteins as LEAPs.

## Results and Discussion

### Dataset: collection and description of the 12 unambiguous LEAP classes

**Figure 1:**
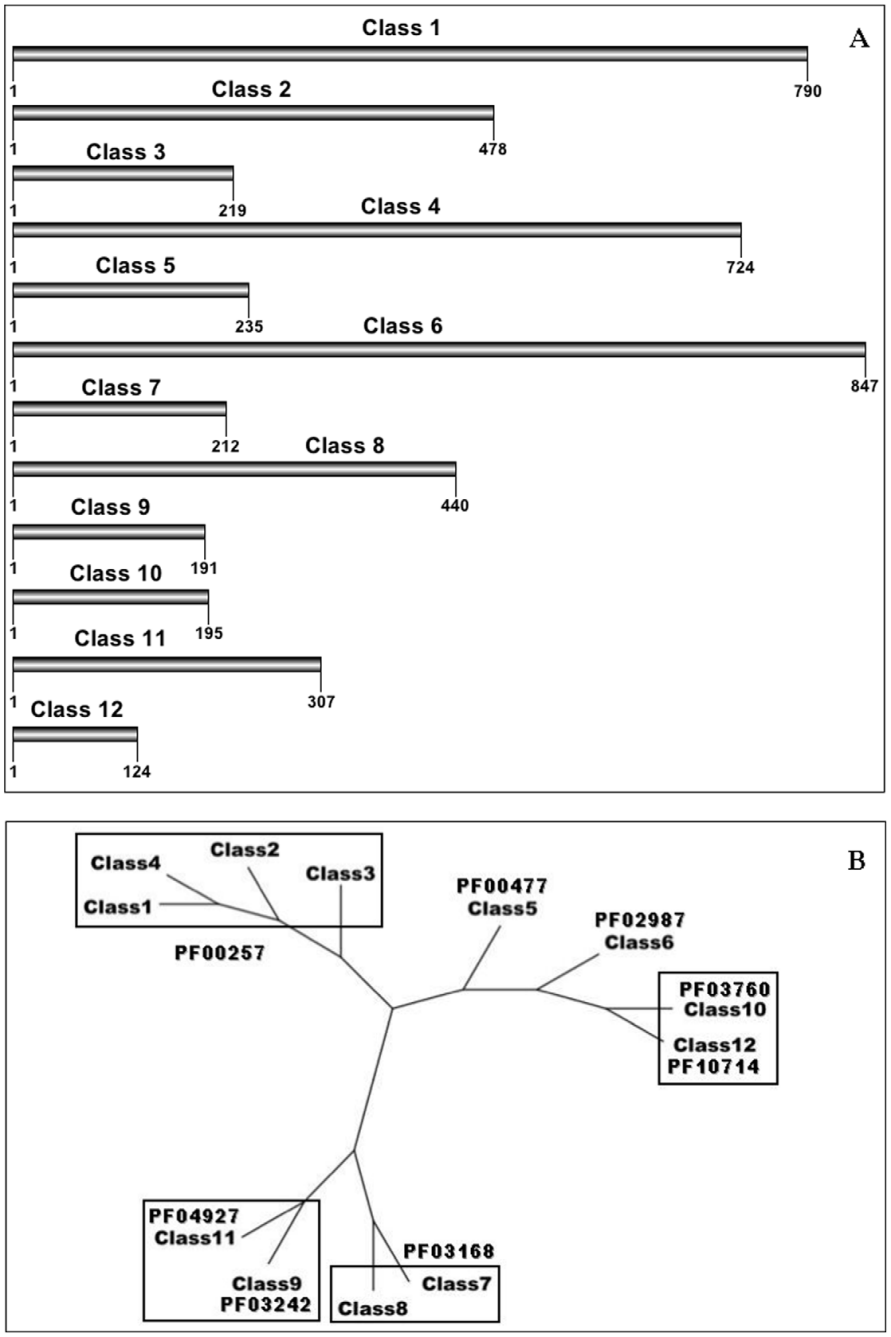
Schematic representation of the 12 LEAP classes. (A) Consensus sequences of the LEAP classes. The way they were obtained introduced gaps (see Material and Methods). Therefore, the lengths indicated in the figure do not reflect real LEAP sequence lengths. (B) Radial phylogram obtained with the 12 LEAP clas consensus sequences.

The dataset consisted of the 710 curated LEAPs sequences available in LEAPdb, which are organized into 12 non-overlapping classes (Table 1). Each class includes a distinct number of LEAP sequences characterized by: (i) a unique amino acid motif matching all sequences of each class without matching any sequence from the other classes. The unique motifs have been selected among different possibilities as the shortest and less degenerate unambiguous signatures of each class. Alignments of sequences of each LEAP class are accessible online (Text S1); (ii) homogeneous PFAM [28], Interpro [29] and CDD [30] annotations. Finally, additional evidence of the rigorous classification of LEAPs into the 12 non-overlapping classes is provided by the high percentage of similarity of the consensus sequences of each LEAP classe (Table 1). Percentages of similarity are equal or above the so-called «twilight zone» [31] in the case of LEAP classes 3, 5, 7, 10, 11 and 12. The average length of consensus sequence (with gaps) for each class varies between 124 (class 12) to 847 (class 6), and the 12 classes organize into three branches on a phylogram (Figure 1).

**Table 1:** Some characteristics of the 12 LEAP classes.

Class	PFAM	Motifs^a^	LEAP number^b^	Length range^c^	Class consensus sequence^d^			
					Total characters	Gap number	% gap	% similarity (35%<CV<60%)
**1**	PF00257	[GS]SSE.[DEG]	145	117–507	790	680	86.1	13.9
**2**	PF00257	S{5,}[ADGV][DES][DEGKT].	65	140–292	478	338	70.7	29.3
**3**	PF00257	DSD$	20	86–179	219	129	58.9	41.1
**4**	PF00257	Motif class 4^e^	63	83–616	724	664	91.7	8.3
**5**	PF00477	G[AG][ENQT].R[AKR][DEQ]	58	83–217	235	145	61.7	38.3
**6**	PF02987	Motif class 6^f^	125	67–742	847	767	90.6	9.4
**7**	PF03168	NPY.{4,}P[IV].[ADEQ]	30	95–181	212	72	34.0	66.0
**8**	PF03168	[AILV].{0,1}NPN.[FIRSVY]	35	153–368	440	329	74.8	25.2
**9**	PF03242	W.{1,3}DP.{1,3}G	64	78–144	191	134	70.2	29.8
**10**	PF03760	[AS].{3,3}[EG][HK].[DE].{3,3}[AT].{4,4}[DEKQ].{3,3}[AT]	68	88–173	195	68	34.9	65.1
**11**	PF04927	(T.GEAL[EH]A)|(PGGVA)	20	159–278	307	133	43.3	56.7
**12**	PF10714	[HY]K.{2,2}[AG]Y	17	71–117	124	58	46.8	53.2

a Meaning of the regular expression syntax used for motifs: «.» = any amino acid; X{n, } = at least n times X; X{n,m} = n to m times X; [XY] = X or Y; [∧XY] = neither X nor Y; (XY) = X followed by Y; X? = X present or not; XY$ = XY at the end; (M1)|(M2) = motif M1 or motif M2 or both.

b Number of sequences in LEAPdb using the motif indicated.

c Amino acid sequences length range of LEAP classes in LEAPdb.

d Consensus sequences of the LEAP classes obtained using Multalin [74]: alignment of all sequences of each LEAP class was performed with a low consensus value (CV) = 35% and a high consensus value = 60% (*i.e.*, above the «twilight zone» [31]) with a PAM matrix (since sequences of each LEAPs class are either distant or not). Gap penalties values (gap open penalty = 2/gap extension penalty = 0/no gap penalty for extremities) were chosen in order to have not stringent conditions for the alignments, thus introducing numerous gaps (see the gaps percentage). This «local - global alignment» of each LEAP class sequences leads to a consensus sequence for each LEAP class, revealing a high level of similarity between those sequences (also much above the «twilight zone»), especially in the case of LEAP classes 3, 5, 7, 10, 11 and 12.

e Motif class 4: STTAPGHY|HKTGTTTS|GGGGIGTG|HS[DR]N?K$|DVE$|LH(TRASHEES)?$|C?TGH$|DKLPGQH$|QQN(KTGCD)?$| RGD$|KEGY$|GHRPQI$|GHNN$|SFKS$|GTHKGL$|SSRDNY$|GQSK$|HRDV$|NDL$.

f Motif class 6: [∧LNP][∧G][ADEGILMQRSTVY] [AEKQRSTY].[KR][AT].[ADENT][∧DP][EGIKLMQST].{1,67}[∧DER][∧AS]K[AD][∧IL][∧N].[∧E]?.{1,6}G?

Two additional datasets were constructed: one includes a series of plant intrinsically disordered proteins (PIDP - 72 sequences from 35 plant organisms) and the other a selection of fully structured proteins (FS - 158 sequences). The size of these datasets is comparable to that of the 12 LEAP classes, and they allow a more comprehensive comparison of LEAP classes structural properties.

LEAPdb provides a large number of physico-chemical properties: number of amino acids (length), molecular weight, isoelectric point, FoldIndex [32], mean (reduced) net charge at pH 7, mean hydrophilicity [33], GRAVY (grand average of hydropathy) [34], mean hydrophobicity (<H>) [35], mean bulkiness [36], mean average flexibility [37], mean molar fraction of accessible residues [38], mean molar fraction of buried residues [38] and mean transmembrane tendency [39] and the percentage of each amino acid. We generated additional data such as combinations of specific amino acids residues, and the relative usage of each amino acid by LEAPs compared to all known proteins (*i.e.*, the Uniprot release of 2010_12) [40]. The physico-chemical properties and the different combinations used in this work are summarized in Table 2.

**Table 2:** Physico-chemical properties and combinations of plain percentages of amino acids of LEAPs.

Physico-chemical properties	
Length	Number of amino acids
MW	Molecular weight
MW/Length	Mean molecular weight
pI	Isoelectric point
Foldindex	Numerical prediction of intrinsic folding propensity [32]
Net charge	Mean net charge at pH 7
Hydrophilicity	Mean hydrophylicity (scale: Hopp & Woods [33])
GRAVY	Grand average of hydropathy (scale: Kyte & Doolittle [34])
Hydrophobicity	Mean hydrophobicity (<H>) (scale: Eisenberg, Schwarz, Wall [35])
Bulkiness	Mean bulkiness (scale: Zimmerman, Eliezer & Simha [36])
Flexibility	Mean flexibility (scale: Bhaskaran & Ponnuswamy [37])
Residues accessibility	Mean value of the molar fraction of 3220 accessible values per residue (scale: Janin [38])
Buried residues	Mean value of the molar fraction of 2001 buried values per residue (scale: Janin [38])
Transmembrane tendency	Mean transmembrane tendency value (scale: Zhao & London [39])

Combinations of amino acids	
D+E	Fraction negative residues
K+R	Fraction positive residues
D+E+K+R	Fraction charged residues
D+E−K−R	Fractional net charge
A+I+L+V	Fraction hydrophobic residues
F+W+Y	Fraction aromatic residues
N+Q	Fraction amide residues
S+T	Fraction alcohol residues
C+W	Fraction rare/absent residues
R+E+S+P	Strong disorder promoting residues
C+F+Y+W	Strong order promoting residues

LEAPs are classified into 12 non-overlapping classes, each LEAP class being clearly characterized by a unique set of properties (Tables 3 and 4). It is well known that proteins with distant sequences can adopt similar 3-D structures, *i.e.*, proteins structures are much more conserved than sequences. This appears rather logical for sequence identity >40%, but it is also true for sequence identity in «twilight zone» range of 20–35% [41]. Such a sequence-structure relationship may be explained by the assumption that protein structure tolerates residue substitutions preserving the sequence hydropathic profile [42]. The finding of consensus sequences of LEAP classes 3, 5, 7, 10, 11 and 12 with percentage of similarity equal to, or above, that of the «twilight zone» (Table 1) confirms the pertinent distribution of LEAPs into the 12 classes. The phylogram presented in Figure 1B illustrates the putative relationship between the 12 LEAP classes. It underlines the «proximity» between LEAP classes, in particular classes 1 to 4 («dehydrins» - PF00257) and between LEAP classes 7 and 8 (PF03168). It is an additional proof of the accuracy of our LEAP classification.

**Table 3:** Binary^a^ representation of the physico-chemical properties distribution among LEAP classes, IDP and FS proteins.

LEAP Class	MW/Length	Fold Index	Mean bulkiness	Mean flexibility	MBR^b^	MAR^c^	MTT^d^	pI	MNC pH 7^e^	Mean hydrophilicity	GRAVY^f^	<H>^g^
**1**	−1	−1	−1	+1	+1	−1	+1	+1	+1	+1	−1	−1
**2**	+1	−1	+1	+1	−1	+1	−1	−1	−1	+1	−1	−1
**3**	+1	−1	−1	+1	−1	+1	−1	−1	−1	+1	−1	−1
**4**	−1	−1	−1	+1	−1	−1	+1	−1	−1	+1	−1	−1
**5**	+1	−1	−1	+1	−1	+1	−1	−1	−1	+1	−1	−1
**6**	−1	−1	+1	−1	−1	+1	−1	+1	+1	+1	−1	−1
**7**	+1	+1	+1	−1	+1	−1	+1	−1	−1	+1	−1	+1
**8**	+1	+1	+1	−1	+1	−1	+1	−1	−1	+1	−1	+1
**9**	+1	+1	+1	−1	+1	−1	+1	+1	+1	+1	−1	−1
**10**	−1	−1	+1	−1	−1	−1	−1	+1	+1	+1	−1	−1
**11**	−1	+1	+1	−1	+1	−1	+1	−1	−1	+1	−1	+1
**12**	−1	−1	+1	+1	−1	+1	−1	−1	−1	+1	−1	−1
**IDP** h	+1	−1	+1	−1	−1	−1	−1	−1	−1	+1	−1	−1
**FS** i	+1	+1	+1	−1	+1	−1	+1	−1	−1	−1	−1	+1

a Values +1 and −1 indicate that the physico-chemical properties considered is upper or lower, than either the overall median value or a reference value (e.g., 7 for pI).

b Mean molar fraction of buried residues.

c Mean molar fraction of accessible residues.

d Mean transmembrane tendancy.

e Mean net charge at pH 7.

f Grand average of hydropathy.

g Mean hydrophobicity.

h Intrinsically disordered proteins dataset.

i Fully structured proteins dataset.

**Table 4:** Binary representation of amino acids usage by LEAPs, IDP and FS proteins compared to the overall proteins contained in Uniprot.

LEAP Class	A	C	D	E	F	G	H	I	K	L	M	N	P	Q	R	S	T	V	W	Y
**1**	−1	−1	−1	−1	−1	+1	+1	−1	+1	−1	+1	−1	−1	+1	−1	−1	+1	−1	−1	+1
**2**	−1	−1	−1	+1	−1	−1	+1	−1	+1	−1	−1	−1	+1	−1	−1	+1	−1	−1	−1	−1
**3**	−1	−1	+1	+1	−1	+1	+1	−1	+1	−1	−1	−1	−1	−1	−1	−1	−1	−1	−1	−1
**4**	−1	−1	−1	−1	−1	+1	+1	−1	+1	−1	−1	−1	−1	+1	−1	−1	+1	−1	−1	+1
**5**	−1	−1	−1	+1	−1	+1	−1	−1	+1	−1	+1	−1	−1	+1	+1	−1	+1	−1	−1	−1
**6**	+1	−1	+1	+1	−1	+1	−1	−1	+1	−1	−1	−1	−1	+1	−1	−1	+1	−1	−1	−1
**7**	−1	−1	+1	−1	−1	−1	−1	+1	+1	−1	−1	−1	+1	−1	−1	+1	−1	+1	−1	−1
**8**	−1	−1	+1	+1	−1	+1	−1	+1	+1	−1	−1	−1	+1	−1	−1	−1	+1	+1	−1	−1
**9**	+1	−1	−1	−1	−1	+1	−1	−1	+1	−1	+1	−1	−1	−1	+1	+1	−1	−1	−1	+1
**10**	+1	−1	−1	+1	−1	+1	+1	−1	+1	−1	+1	+1	+1	+1	−1	−1	+1	−1	−1	−1
**11**	+1	−1	+1	−1	−1	+1	−1	−1	−1	−1	−1	−1	−1	+1	−1	−1	+1	+1	−1	−1
**12**	+1	−1	+1	+1	−1	+1	+1	−1	+1	−1	−1	−1	+1	+1	−1	−1	+1	−1	−1	+1
**IDP** a	−1	−1	−1	+1	−1	+1	+1	−1	+1	−1	−1	−1	+1	−1	−1	−1	−1	−1	−1	−1
**FS** b	−1	−1	+1	−1	−1	+1	+1	−1	−1	−1	−1	+1	−1	−1	−1	−1	+1	+1	+1	+1

Values +1 and −1 indicate that the median value of the ratio (% amino acid considered in LEAP/% amino acid considered in Uniprot) is upper or lower than 1 (Figure 3 and Figures S4, S5, S6 and S7).

a Intrinsically disordered proteins dataset.

b Fully structured proteins dataset.

### Computation of the physico-chemical properties of the 12 LEAP classes

Mean values are values normalized to chain length. They are uniformly more predictive than total values for significantly correlated parameters [43].

#### a. Reduced molar mass (MW/length)

**Figure 2:**
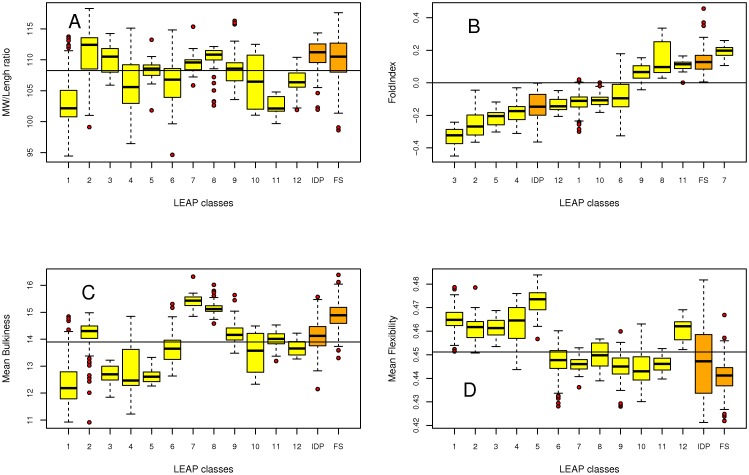
Boxplot representation of MW/length ratio, FoldIndex, mean bulkiness and mean flexibility of the 12 LEAP classes, IDP and FS proteins. The line indicates either the mean or the median value calculated for the 12 LEAP classes. In the case of the FoldIndex, the line corresponds to 0. (A) MW/Length. (B) FoldIndex. Data are presented as a graded scale for a better comparison of classes *vs.* IDP and FS datasets. (C) Mean bulkiness. (D) Mean flexibility.

This value is the molar mass of a polypeptide chain divided by its number of amino acids. Thus, it corresponds to the mass for the same length of the α-carbon backbone. Using this scale, one can discriminate between «light» (median MW/length ratio below the overall median for classes 1, 4, 6, 10, 11, 12) and «heavy» LEAP classes (median MW/length ratio above the overall median for classes 2, 3, 5, 7, 8, 9) (Figure 2A).

#### b. FoldIndex

This parameter reflects the propensity of a protein to be an intrinsically disordered protein (IDP) or to contain intrinsically disordered regions (IDR). In Figure 2B, LEAP classes are presented in ascending FoldIndex order in abscissa. Four classes (7, 8, 9 and 11) have positive FoldIndex values indicating a high content of structured regions. Indeed, class 7 (PF03168) contains the two LEAPs whose 3D structures have been determined (LEAP acc. # O03983 corresponding to PDB code 1XO8 - coded by AT1G01470 and LEAP acc. # 1YYC corresponding to PDB code 1YYC - coded by AT2G46140) [24]. As expected, a high number of LEAPs (classes 1 to 6, 10, 12) display negative FoldIndex values that are indicative of IDPs.

#### c. Mean bulkiness

This parameter takes into account the van der Waals volumes of amino acid residues. LEAP classes 7 and 8 are the bulkiest while among the less bulky one finds LEAP classes 1, 3, 4 and 5 (Figure 2C).

#### d. Mean flexibility

Protein flexibility is dependent on the abundance of residues with short and unconstrained lateral chains. LEAP classes are clearly divided in two groups: classes 1 to 5 and class 12 exhibit higher mean flexibility and classes 6 to 11, lower mean flexibility (Figure 2D).

#### e. Isoelectric point and mean net charge at pH 7 <R>

LEAP classes are either acidic (classes 2, 3, 5, 7, 8, 11 and 12), neutral (classes 4 and 6) or basic (classes 1, 9 and 10). Classes 7, 8, 9 and 11 have the most extreme isoelectric point values (Figure S1A). This classification mirrors the mean net charge at pH 7 (Figure S1B). The fractional net electrostatic charge at neutral pH (*i.e.*, the number of Arg+Lys residues minus the number of Asp+Glu residues, normalized by protein chain-length), also gives the same class profile (data not shown).

#### f. Mean hydrophilicity

Most classes display a high positive hydrophilicity, which is a landmark of LEAPs (Figure S1C). However, class 7, which contained the two LEAPs with established 3D structures, has a near null median hydrophilicity value. Mean hydrophilicity seems to characterize disorder: LEAPs in class 7 have a slightly negative value comparable to that of FS proteins, while all other LEAP classes have a pronounced positive value like IDP.

#### g. Mean molar fraction of buried residues, mean molar fraction of accessible residues and mean transmembrane tendency

The profiles obtained for mean molar fraction of buried residues (Figure S2A) and mean transmembrane tendency (Figure S2C) exhibit identical distribution, which are logically the opposite of the distribution observed for accessible residues (Figure S2B), except for classes 4 and 10. All transmembrane tendency values are negative, which suggests that no LEAP should be integral membrane protein in the native state.

#### h. Grand average of hydropathy (GRAVY) and mean hydrophobicity <H>

Classification using these two parameters results in an identical distribution of LEAP classes, reflecting the global hydrophobicity of the proteins. However, it must be noted that LEAP classes 7 and 8 have GRAVY values close to zero (Figure S3A) and positive <H> values (Figure S3B). This result is likely linked to the fact that these two classes are included in PF03168 and class 7 comprises LEAPs with 3D structures.

#### i. Binary distribution of the physico-chemical properties within LEAP classes

All the physico-chemical parameters described above were also expressed in a binary mode (Table 3), in order to reflect the distribution of each class with reference to the overall median or a reference value (e.g., 7 for pI). This analysis clearly illustrates the unique distribution pattern of physico-chemical properties among LEAP classes, highlighting common and distinctive features.

### LEAP amino acids analysis

#### LEAP amino acids usage

The percentage of each amino acid was calculated for each LEAP class. This value was then divided by the percentage of each amino acid found in release 2010_04 of UniProtKB/Swiss-Prot [40]. This ratio thus describes the frequency of usage of each amino acid by LEAPs. In other words, a value of 1 means the usage of a given amino acid is the same as its usage by all proteins contained in Uniprot.

**Figure 3:**
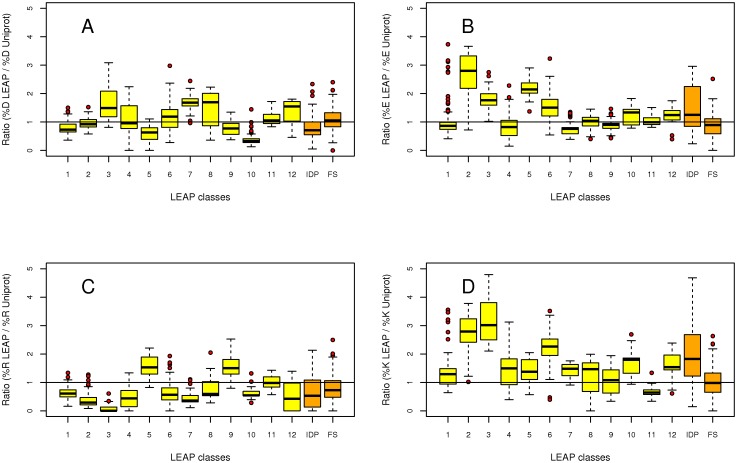
Boxplot representation of charged amino acid usage by the 12 LEAP classes, IDP and FS proteins. The percentage of each amino acid was first calculated for each LEAP class. This value was then divided by the percentage of each amino acid found in the release 2010_04 of UniProtKB/Swiss-Prot [41]. This ratio thus describes the frequency of usage of each amino acid by LEAPs. The line corresponds to a ratio equal to 1. (A) Ratio for Asp. (B) Ratio for Glu. (C) Ratio for Arg. (D) Ratio for Lys.

*Charged amino acids (Asp, Glu, Arg and Lys):* Asp and Glu residues are almost equally used, although there is a preference for Asp in LEAP class 7, and a preference for Glu in LEAP classes 2, 3, 5, and 6 (Figure 3A & B, respectively). All LEAP classes (with the exception of class 11) use much more Lys than most proteins (Figure 3D). On the contrary, Arg is generally less used (with the exception of classes 5 and 9). The use of these amino acids can also be represented as the fractional content of negatively or positively charged residues [41], *i.e.*, the number of [D+E] or the number of [K+R] residues, respectively, normalized by protein chain-length (Figure S8A & S8B). This reveals that the classes most enriched in acidic residues are also highly enriched in basic residues, and conversely, except for class 12.

*Gly:* The smallest residue is largely over-represented in classes 1, 3, 4, 5, 10 and 12 (Figure S4A). Conversely, it is under-represented in classes 2 and 7, the latter likely comprising natively 3-D structured LEAPs. Since there is no obvious correlation between Gly usage and FoldIndex (Figure 2B), the accumulation of this small residue in LEAPs does not explain their propensity for structural disorder.

*Cys:* is almost absent in LEAP classes 3, 5, 10 and 12, or largely under-represented for the others (Figure S4B). The occurrence of intra-chain disulfide bridges (or inter-chain if LEAPs adopt quaternary structure) is therefore either unlikely or impossible for LEAPs.

*Asn* and *Gln:* Asn is largely under-represented in all LEAP classes. Conversely, Gln is over-represented with the exception of classes 7, 8 and 9 (Figure S4C & D).

*Phe, Tyr and Trp:* The overall representation of aromatic amino acids was found to be very low (Figures S5A to S5C): Phe is absent from LEAP classes 10 and 12 while LEAP classes 3, 10 and 12 contain no Trp. However, LEAP class 9 is a noticeable exception since the motif characterizing this class contains one Trp.

*His:* This residue has a very peculiar distribution since it is highly represented in classes 1 to 4. Class 3 uses His up to 6 times more than the average usage in all UniProt proteins. His is also over-used by LEAP class 10 (Figure S5D). His seems an amino acid characteristic of IDP.

*Non-polar hydrophobic amino acids (Ala, Leu, Ile and Val):* They are generally under-represented by all LEAPs with some exceptions (Figures S6A to S6D). Ala, the smallest of the four, is the preferred amino acid of this type and is over-used by LEAPs belonging to classes 6, 9, 10 and 11. Therefore, this category of amino acids is likely not responsible for the low GRAVY and <H> values of LEAPs (Figure S3). Since hydrophobic residues mainly contribute to the hydrophobic core of natively folded proteins, this can explain why LEAPs establish few interactions with other proteins in their intrinsically disordered unfolded state, as in the case of LEAM [18].

*Ser* and *Thr:* LEAPs generally use less Ser than other proteins, an exception being class 9 (Figure S7A). On the contrary, they use Thr much more frequently, up to twice in the case of classes 1 and 4 (Figure S7B). This residue is however less represented in classes 2 and 3.

*Met* and *Pro:* LEAP classes 3, 5 and 6 have a very low Pro content. However, there is no obvious rule of usage for these two amino acids (Figure S7C & D, respectively).

#### Binary representation of amino acids usage by LEAPs compared to the overall proteins contained in Uniprot

All previous results are summarized using a binary representation (Table 4).

### Normality of the distributions and its consequences

**Figure 4:**
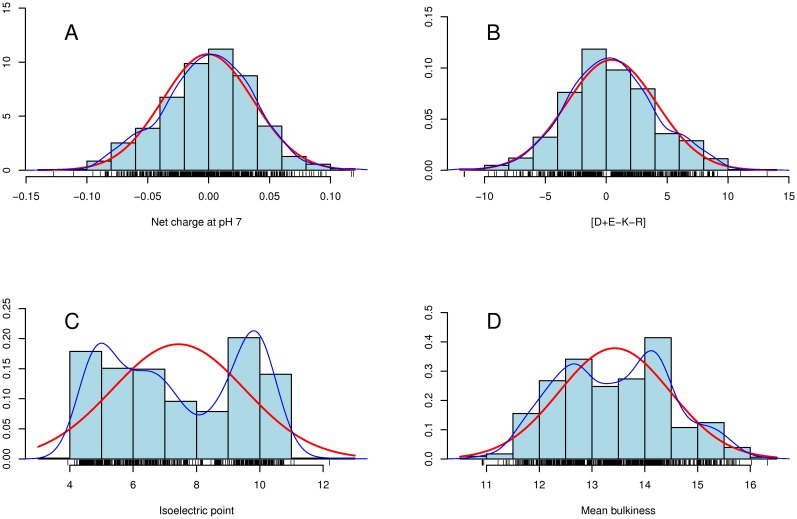
Some normal and non normal distributions of the variables for the 710 LEAP contained in LEAPdb [**8**] **.** The red line corresponds to the normal distribution associated to the data whereas the blue line corresponds to the estimated density curve. (A) and (B) Normal distributions for mean net charge at pH 7 and [D+E−K−R] combination. (C) and (D) Non normal distributions for isoelectric point and mean bulkiness.

The statistical univariate analysis showed that only mean net charge at pH 7 and the [D+E−K−R] combination could be considered as normal distributions (Figures 4A & 4B, respectively). All other variables were considered to deviate too much from the normal distribution, according to the *Shapiro* and *Kolmogorov-Smirnov*'s normality tests (all p-values were less than 0.001, results not shown) and graphical visualizations such as histograms and Q-Q plots (not shown). However, even for the variables that could be considered as normal on the whole, the distribution within each class could not be considered as normal, as is shown in Figure S9.

Therefore, all subsequent statistical analyses were performed with non parametric tests that do not require the normality assumption usually used in parametric tests. For instance, to test the correlation between variables, we used *Spearman*'s r instead of the classical *Bravais-Pearson*'s r coefficient.

### Principal Component Analysis (PCA)

**Figure 5:**
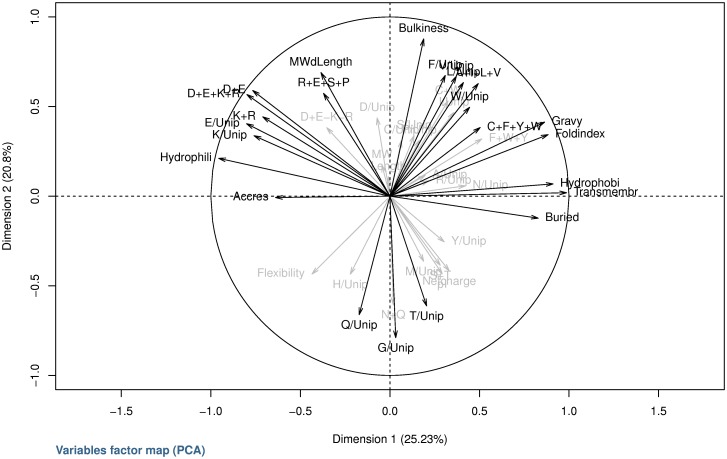
The main projection of the variables on the first two axis of the PCA. This is the correlation circle of the projection of the 45 variables using the first two components of the PCA. Axis I is horizontal and axis II is vertical. The most- contributing variables are in black, the others in grey.

In order to have a global visualization of the relations between the variables, we performed a PCA on all 43 quantitative variables and all 710 proteins. The inertia of the first two axis sums up to 48% (according to *Cattell*'s scree test, one should retain 3 axes, thus obtaining 57% of inertia). In Figure 5, the variables in grey have a lower contribution to the axis, compared to the other variables (cos^2^<0.01). In Figure S10, one can see the relative positions of the center of the classes and their confidence ellipse altogether with the variables.

The first axis can be interpreted as revealing the opposition between the variables GRAVY, FoldIndex, hydrophobicity, mean transmembrane tendency, mean molar fraction of buried residues versus hydrophilicity and the high values of (% Glu LEAP/% Glu Uniprot), [D+E+K+R] combination (Figure S11A), [D+E] combination, (% Lys LEAP/% Lys Uniprot), [K+R] combination and mean molar fraction of accessible residues altogether with the opposition of classes 7, 8 and 9 against classes 2 and 3. The first axis can thus be defined as the opposition of the charge versus the hydrophobicity tendency.

The second axis shows mean bulkiness, MW/Length, (% Phe LEAP/% Phe Uniprot), (% Val LEAP/% Val Uniprot), (% Leu LEAP/% Leu Uniprot) and [A+I+L+V] combination (Figure S11C) as opposed to (% Gly LEAP/% Gly Uniprot), (% Gln LEAP/% Gln Uniprot), (% Thr LEAP/% Thr Uniprot) and [N+Q] combination (not shown), this time in conjunction with classes 7 and 8 as the most distant from classes 1 and 4. The third axis separates mean net charge at pH 7 and isoelectric point versus [D+E−K−R] combination (Figure S11B) and (% Asp LEAP/% Asp Uniprot).

### Correlation analysis of the different variables

Because of the non-normality of the distribution of all variables, the bivariate analysis used *Spearman*'s coefficient of correlation instead of the classical *Bravais-Pearson* coefficient of linear correlation. It revealed several sets of highly inter-correlated variables. We decided to apply a threshold of 0.75, in absolute value, for all variables in order to build sets of variables taking in account all the correlation coefficients (Table 5). This approach provides a better visualization of the correlation among variables than the classical full matrix of correlation coefficient with its 43 lines and columns. The next best correlation coefficient after those shown in Table 5 is 0.603 and the other high correlation coefficients are also listed in the table. From the strong correlations that were found, one may globally consider: (i) net charge at pH 7 and the [D+E−K−R] combination as equivalent but of opposite signs; (ii) GRAVY, FoldIndex, mean hydrophobicity <H>, mean molar fraction of buried residues and mean transmembrane tendency as expressing nearly the same property; (iii) [D+E+K+R] combination, mean hydrophylicity and the ratio (% Glu LEAP/% Glu Uniprot) are very strongly related; (iv) mean bulkiness is equivalent to (% Gly LEAP/% Gly Uniprot) but with an opposite sign; (v) mean flexibility and [A+I+L+V] combination can also be considered as equivalent.

**Table 5:** Groups of highly inter-correlated variables and other high correlation coefficients among variables using Spearman's r.

Groups of highly inter correlated variables			
Count	Highest r	Lowest r	Variables
5	0.984	0.805	GRAVY; FoldIndex; Mean hydrophobicity; Mean molar fraction of buried residues; Mean transmembrane tendency
4	0.929	0.798	[D+E+K+R] combination; [D+E] combination; Mean hydrophilicity; (% Glu LEAP/% Glu Uniprot)

Other high correlation coefficients among variables		
Spearman's r	Variable 1	Variable 2
−0.995	[D+E−K−R] combination	Mean net charge at pH 7
0.972	pI	Mean net charge at pH 7
−0.962	pI	[D+E−K−R] combination
0.962	[C+F+Y+W] combination	[F+W+Y] combination
−0.882	(% Gly LEAP/% Gly Uniprot)	Mean bulkiness
0.876	[N+Q] combination	(% Gln LEAP/% Gln Uniprot)
−0.842	[A+I+L+V] combination	Mean flexibility
0.829	Mean hydrophilicity	[K+R] combination

We also computed the classical hierarchical clustering that follows a PCA (*Ward*'s method applied to the Euclidean distances) for the LEAPs using their coordinates on the first principal components of the PCA. Most LEAPs of each class are aggregated at a low hierarchical level as shown in Figure S12. However, in the full dendrogram with all 710 LEAPs (not shown because details are not easily seen) the clusters at the highest levels contain a few LEAPs from distinct classes, probably indicating that the distances between the LEAPs induced by the ACP do not discriminate the classes.

### Some general rules for LEAP classes

Cys, Asn, Leu, Phe, Trp are largely less represented in, or absent from all LEAP classes relative to all proteins in Uniprot. Moreover, most LEAP classes use less Ile, Tyr and Val. A general characteristic of LEAPs is the use of a smaller subset of the 20 amino acids.

The preferential use of Lys over Arg is almost systematic in the 12 LEAP classes. In the case of proteins from hyperthermophiles (enriched in Lys at the expense of Arg), it has been shown that Lys (but not Arg) exhibits significant residual dynamics in the folded states of proteins. This makes the entropic cost to fold Lys-rich proteins more favourable that to fold Arg-rich ones [44]. Preference of Lys over Arg could thus provide additional thermal stabilization of LEAPs via an entropic mechanism.

It has been demonstrated that members of LEAP class 1 interact with membrane phospholipids [45]. It was also shown that a LEAP class 10 (PF03760) from *Arabidopsis thaliana* (At2g35300) does not stabilize membrane protein, but could possibly modulate the membrane stability as a function of the membrane composition [46]. Since the variety of cellular membrane compositions is limited, it is unlikely that all LEAP classes function via interactions with membrane phospholipids. This supports the possibility that LEAPs have a variety of alternative functions and/or interactions with numerous cellular partners.

LEAP classes 2 and 3 are the most hydrophobic. They largely use Lys (up to 3 times more than typical inUniprot) instead of Arg, and Glu (also up to 3 times) instead of Asp. LEAP class 2 is the sole class using less Gly than typical while LEAP class 3 uses His up to 6 times more.

LEAP class 7 is rather peculiar, not only because it comprises the only two LEAPs with established 3D structure, but also because it displays signatures often diverging from the other classes. The results indicate that LEAPs from class 7 have a high content of structured regions.

Classes 7, 8, and 11: (i) have positive FoldIndex values and, logically, a low flexibility, indicating a high content of structured regions; (ii) are acidic and have the most extreme isoelectric point values, logically correlated to their mean net charge at pH 7. The fractional net electrostatic charge at neutral pH (*i.e.*, the number of Arg+Lys residues minus the number of Asp+Glu residues, normalized by protein chain-length) fits with the same classes profile (data not shown); (iii) are the bulkiest; (iv) exhibit the highest mean molar fraction of buried residues and the highest mean transmembrane tendency; (v) have near zero GRAVY values and zero or positive <H> values.

To test the presence of significant differences between LEAP classes, we performed the non parametric *Kruskal-Wallis*' test instead of the classical one-way *ANOVA* (analysis of variance). All quantitative variables appeared significantly different (all p-values <0.001, results not shown) for the «class» factor, thus demonstrating on statistical grounds the relevance of the definitions of the 12 LEAP classes. Due to the great number of variables, it is not possible to provide here all the conclusions pertaining to the computations following the *Kruskal-Wallis*' test and the subsequent *Nemenyi*'s post-hoc tests for all variables. We therefore focus on the most important results. Concerning LEAP physico-chemical properties, it should be noted that: (i) LEAP classes 7, 8, 9 and 11 have globally a marked positive FoldIndex (Figure 2B) which discriminates them from all the other classes; (ii) LEAP classes 1, 3, 4 and 5 show lower values of mean bulkiness (Figure 2C); (iii) the mean flexibility values separate LEAP classes 1 to 5 and 12 from all the remaining classes (Figure 2D); (iv) the isoelectric point values show that LEAP classes 9 and 10 are being high valued and LEAP classes 11 and 12 are being low valued (Figure S1A).

Looking at the amino acid composition: (i) For Gly, LEAP classes 1, 3, 4 and 5 are highly enriched (Figure S4A); (ii) even for the amino acids Cys (Figure S4B) and Phe (Figure S5A), that are strongly under-represented (or completely absent from some LEAP sequences), there is a significant difference between the 12 LEAP classes; (iii) LEAP class 3 has a remarkable His content: 6 times more on average than all proteins contained in Uniprot (Figure S5D); (iv) LEAP classes 2 and 3 have a very high proportion of charged residues (Figure S11A) while only LEAP classes 9 and 10 clearly have a global negative net charge (Figure S11B); (v) LEAP classes 2, 5 and 9 are highly enriched in disorder promoting residues (Figure S8C); (vi) LEAP class 11 is unique with its high content of hydrophobic residues (Figure S11C).

LEAP classes 1 to 4 display distinct values for the variables, which implies that the definitions of the «class» are relevant.

### Disorder and structure in LEAP classes

Natively folded proteins and IDPs occupy non-overlapping regions in the mean net charge (<R>) *vs.* mean hydrophobicity (<H>) plots, with natively IDP localized below a zone delimited by the following equation: <H> normalized = 0,560 <R>+0,645. In the original article of Uversky *et al.* [21], graphics and equations are presented under the equivalent form <R> = 2,785 <H>−1,151, with IDP above the delimitation line. It has been shown that the combination of low mean hydrophobicity (*i.e.*, less driving force for protein compaction) and relatively high mean net charge (*i.e.*, charge–charge repulsion) is important for the absence of compact structure in proteins under native conditions [47].

**Figure 6:**
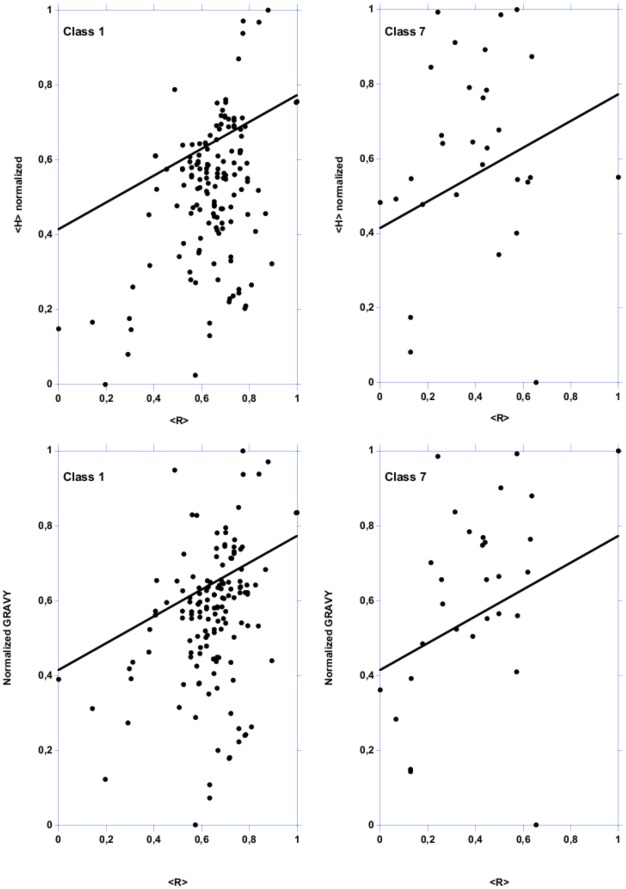
Mean normalized hydrophobicity (<H>) *vs.* mean net charge (<R>) plot and mean normalized GRAVY *vs.* mean net charge (<R>) plot for LEAP classes 1 and 7. The two areas are delimited by the following equations: <H> normalized = 0,560 <R>+0,645 and normalized GRAVY = 0,359 <R>+0,413. The line indicates the boundary between folded (above) and unfolded (below) polypeptide chains.

Most LEAPs from class 1 are localized below the line while most LEAPs of class 7 are localized above that line (Figure 6), confirming that LEAPs in class 1 are intrinsically unstructured while LEAPs in class 7 are natively folded. The results also indicate that LEAP classes 2, 5, 6, 8 and 10 mostly comprise IDPs (Figures S13 and S14). For other LEAP classes, it is more difficult to reach a conclusion because either there is an equal distribution in both areas, or the number of LEAPs is too small. Although this type of plot gives an indication about the propensity of amino acids segments to be unstructured, the actual protein structure depends on the overall polypeptide chain.

However, these results are confirmed by plotting the charge - hydropathy distribution, *i.e.*, normalized GRAVY *vs.* <R> (Figure 6 for LEAP classes 1 and 7, not shown for other LEAP classes). The two areas are delimited by a line following the equation: normalized GRAVY = 0,359 <R>+0,413. Moreover, LEAP classes 7, 8, 9 and 11 have positive FoldIndex values that indicate a high content of structured regions (Figure 2B), confirming the observed charge-hydrophobicity distributions.

It is now well established that under physiological conditions a substantial number of proteins, the so-called IDPs, either completely lack stable structure or contain long unstructured domains [48]–[49]. IDPs are frequently involved in cellular regulatory processes (e.g. in signal transduction or in the regulation of gene expression [50]–[51]), demonstrating a structural transition (at least an increase of secondary structure) upon binding to their nucleic acids or protein targets. Therefore, structural disorder may confer functional advantages as, for example, the binding of an IDP to multiple partners, which makes sense for LEAPs that are expected to be versatile protectants during anhydrobiosis. IDPs are depleted in order-promoting amino acids (Trp, Tyr, Phe, Ile, Leu, Val, Cys and Asn) and enriched in disorder-promoting, amino acids (Ala, Arg, Gly, Gln, Ser, Glu, Lys, Pro) [52]. LEAP amino acid usage clearly indicates that this rule applies for most LEAPs. The study of Price *et al.* [43] has shown that the frequencies of Ala, Gly and Phe positively correlate with successful crystal-structure determination whereas the frequencies of Glu and Lys negatively correlate. This could be linked to the fact that Ala and Gly have the lowest side-chain entropies, whereas Lys and Glu have among the highest side-chain entropies. Because of their amino acid composition and IDP features, it is therefore not surprising that LEAPs are almost absent from protein structure databases.

It is known that the planar geometry and the charge delocalization of Phe, Tyr, Trp and Arg facilitate different types of interactions with a large number of other residues [53]. Therefore, the lack of, or low representation of such residues could partly contribute to the non-structured character of LEAPs. Some amino acid combinations may discriminate between ordered and disordered polypeptides [54]. For example, LEAP classes 2, 5 and 9 have the highest [R+E+S+P/length] ratio, (*i.e.*, the strongest disorder promoting residues [55], Figure S8C) whereas LEAP class 7 has a lower ratio. LEAP classes 7, 8 and 9 have the highest [C+F+Y+W/length] ratio (*i.e.*, the strongest order promoting residues, Figure S8D). As a whole, most LEAP classes comprise a majority of IDPs, which can be clearly corroborated with their physico-chemical properties profile and their amino acid composition and combinations.

**Figure 7:**
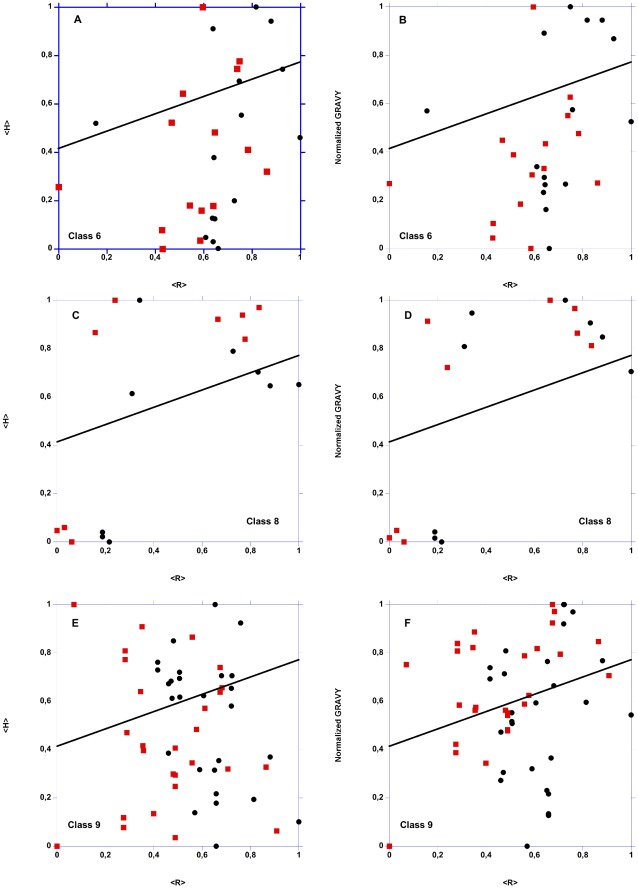
Mean normalized hydrophobicity (<H>) *vs.* mean net charge (<R>) plot and mean normalized GRAVY *vs.* mean net charge (<R>) plot for full-length and mature LEAP classes 6, 8 and 9. The two areas are delimited by the following equations, respectively: <H> normalized = 0,560 <R>+0,645 and normalized GRAVY = 0,359 <R>+0,413. The line indicates the boundary between folded (above) and unfolded (below) polypeptide chains. A & B: LEAP class 6; C & D: LEAP class 8; E & F: LEAP class 9. Circles: full-length LEAPs. Squares: mature LEAPs (*i.e.*, without the signal peptide).

Since proteins targeted to the secretory pathway, mitochondria or plastids are generally synthesized as precursors with N-terminal pre-sequences cleaved upon import to yield mature proteins, disorder predictions could be possibly biased by the pre-sequences. We therefore used a battery of signal and targeting signal predictors to identify LEAPs that could be subjected to such maturation, and to identify their putative pre-sequence cleavage site. Interestingly, 51 proteins were clearly predicted with a cleaved pre-sequence, 15 belonging to LEAP class 6, 9 to class 8 and 26 to class 9. Among these proteins, 40 were predicted to be targeted to mitochondria, 5 to plastids and 6 to the secretory pathway. The IDP features of the corresponding LEAPs were then revaluated with the putative mature protein sequences. Figure 7 shows the <H> *vs.* <R> plots and the normalized GRAVY *vs.* <R> plots for full-length LEAPs and mature LEAPs (*i.e.*, without their peptide sequences). In the case of class 6, the mature form appears enriched below the line (delimiting structured *vs.* non-structured proteins), especially in the case of the GRAVY *vs.* <R> plot (Figure 7B). This indicates that the frequency of disorder in this class is slightly overestimated. For class 8 LEAPs, there is no obvious difference between the repartition of precursor and mature sequences (Figure 7 C, D). In the case of class 9, there is an enrichment of mature forms below the line in the <H> *vs.* <R> plot (Figure 7E), but the opposite is found for the GRAVY *vs.* <R> plot (Figure 7F). We also performed a comparative analysis of the 51 LEAP precursors and their mature sequences with FoldIndex. This confirmed that the pre-sequences had indeed no influence on the disorder predictions for LEAPs in classes 8 and 9, and only slightly overestimated the disorder prediction for the class 6 proteins (data not shown). Overall, the presence of these putative 51 pre-sequences in the dataset does not have a significant influence on the IDP analyses. This is likely due to the relatively short length (25±14 amino acids, SD) of the putative pre-sequences. Although taking in account the fact that the cleavage of a pre-sequence is crucial for structural and functional analyses of specific proteins (e.g. mitochondrial proteins), this appears of less concern for large-scale computational analysis of sequences. Since this concerns only a small fraction of amino acids in the LEAP dataset, and also because experimental data are lacking, this was not taken into account in the other computational analyses.

A survey of the literature identified 30 LEAPs, essentially from plants, which have been subjected to experimental structural characterization, mainly secondary structure analysis under various conditions, as indicated in Table 6 (and references herein). All classes except 3 and 8 comprise one or several proteins with established structural features. Apart from class 7 which is represented by the two proteins with established 3D-structure (LEA14 and LEA2R), all proteins display low levels of secondary structure in the hydrated state (Table 6), which agrees well with the predicted IDP character of LEAPs. Most of them displayed structural transitions with increased levels of secondary structure (α-helix, β-sheet) upon various conditions such as the addition of trifluoroethanol (an α-helix promoting agent), detergents or lipid vesicles, or upon drying (Table 6). Such propensity of these LEAPs to acquire higher structural order is likely related to their functional role as protein and/or membrane stabilizers [16]. For proteins that display only modest structural transitions, the possibility remains that only a part of the polypeptide is affected by major transitions, as shown in the case of the K-segment of DHN1 [20]. Because the number of proteins in this experimental dataset is very low (2–5 LEAP per class), and also because the analyses were performed with different methods and conditions, it is not possible to establish significant correlations between LEAP classes and structure of the proteins. There is therefore a need for the analysis of LEAPs to be performed in a comparative fashion (e.g. 5 LEAPs in ref. [63]) to standardize the results and uncover structure-function relationships.

**Table 6:** Compilation of secondary structure data available for LEAPs.

ACCESSION	CLASS	STRUCTURAL FEATURES AND TRANSITIONS	METHODS	SPECIES	REF.
P_201441	1	12% PII helix; no α-helix induction with TFE	CD	*Arabidopsis thaliana*	[56]
AAK00404	1	15% α-helix; 30% α-helix with TFE; no structural change with lipid vesicles	^1^H-NMR, CD	*Arabidopsis thaliana*	[57]
CAA33364	1	Largely unstructured; 9–10% α-helix with lipid vesicles or SDS (attributed to K segment, a 15 aminoacid peptide)	CD	*Zea mays*	[20], [58]
NP_850947	2	15% α-helix; 30% α-helix with TFE; no structural change with lipid vesicles	^1^H-NMR, CD	*Arabidopsis thaliana*	[57]
NP_850947	2	12% PII helix; 20–30% α-helix with TFE;	CD	*Arabidopsis thaliana*	[56]
NP_173468	2	5% α-helix, 15% PII helix; 50% α-helix with TFE;	CD	*Arabidopsis thaliana*	[56]
ADK66263	2	Largely unstructured, presence of PII helix at low temperature; 3–10% α-helix and 50–75% β-sheet with lipid vesicles; Structural transitions stimulated by phosphorylation, Zn	FTIR	*Thellungiella salsuginea*	[59], [60], [61]
AEE78733	4	5% α-helix, 12% PII helix; 20–30% α-helix with TFE;	CD	*Arabidopsis thaliana*	[56]
AAA18834	4	27% PII helix at 12°C, decrease to 15% at 80°C; no α-helix induction with TFE or SDS	CD	*Glycine max*	[62]
CAA77508	5	1% α-helix and 18% β-sheet; increase to 38% α-helix upon drying	CD	*Arabidopsis thaliana*	[63]
NP_190749	5	3% α-helix and 19% β-sheet; increase to 23% α-helix upon drying	CD	*Arabidopsis thaliana*	[63]
ABB13462	5	33% α-helix; increase to 56% α-helix upon drying	FTIR	*Medicago truncatula*	[15]
AAB68027	5	14% PII helix, 8% α-helix; 30% α-helix with TFE;	CD	*Glycine max*	[64]
CAA36323	5	13% α-helix and 17% β-sheet; 29% α-helix with TFE	CD	*Triticum aestivum*	[65]
NP_181782	6	10% α-helix in the hydrated state, 65% α-helix in the dry state	CD	*Arabidopsis thaliana*	[66]
NP_181781	6	17% α-helix in the hydrated state, 57% α-helix in the dry state	CD	*Arabidopsis thaliana*	[66]
NP_175678	6	27% α-helix and 15% β-sheet in the dry state; α-helix formation is favored by lipid vesicles in the hydrated and dry states. β-sheet is attributed to aggregation.	CD, FTIR	*Arabidopsis thaliana*	[67]
CAF32327	6	3% α-helix and 14% β-sheet in the hydrated state; 70% α-helix with TFE or in the dry state	CD, FTIR	*Pisum sativum*	[18]
AAL18843	6	Largely unstructured; α-helix and coiled coil formation upon drying	CD, FTIR	*Aphelenchus avenae*	[68]
O03983	7	Structured protein: 9% α-helix and 41% β-sheet	NMR	*Arabidopsis thaliana*	[24]
NP_182137	7	Structured protein: 18% α-helix and 42% β-sheet	NMR	*Arabidopsis thaliana*	
ACJ46652	9	3% α-helix and 25% β-sheet; 40% α-helix with TFE or upon drying	Sync rad, CD, ^1^H NMR, FTIR	*Lotus japonicus*	[69]
AAC61808	10	15% β-sheet in the hydrated state, 35% β-sheet with lipid vesicles	CD	*Arabidopsis thaliana*	[46]
ABB72365	10	25% α-helix; 90% α-helix with TFE, SDS or upon drying	CD, FTIR	*Glycine max*	[70]
Q01417	10	15–17% α-helix and 15–16% β-sheet; 36% α-helix upon drying	CD, FTIR	*Glycine max*	[71]
AAD09208	10	Largely unstructured	CD	*Glycine max*	[72]
AAF21311	11	33% α-helix, 18% β-sheet; increase to56% α-helix, 25% β-sheet upon drying	FTIR	*Medicago truncatula*	[15]
AAF21311	11	FTIR spectroscopy	CD	*Medicago truncatula*	[73]
NP_179892	12	3% α-helix and 20% β-sheet; increase to 25% α-helix upon drying	CD	*Arabidopsis thaliana*	[63]
NP_565548	12	3% α-helix and 16% β-sheet; increase to 20% α-helix and 31% β-sheet upon drying	CD	*Arabidopsis thaliana*	[63]
AAS47599	12	1.5% α-helix and 22% β-sheet; increase to 19% α-helix upon drying	CD	*Arabidopsis thaliana*	[63]
DN776754.1	NC	Largely unstructured, presence of PII helix at low temperature; 5–30% α-helix and 30–80% β-sheet with lipid vesicles; Structural transitions stimulated by phosphorylation, Zn	FTIR	*Thellungiella salsuginea*	[59], [60], [61]

NC, not classified in LEAdb; CD, Circular dichroism; NMR, Nuclear magnetic resonance; FTIR, Fourier transformed infrared spectroscopy; Sync rad, Synchrotron radiation.

In conclusion, this work provides, to our knowledge, the most exhaustive computational analysis of the physico-chemical properties of LEAP amino acid sequences. It is based on a new unambiguous and rigorous classification of LEAPs into 12 non-overlapping classes, and the computational analyses provides a solid basis to develop software to predict whether any new sequence is a LEAP or not, an aspect currently under progress. We hope that this classification as well as the term «class» will be adopted instead of the various «groups» typically employed in the past.

The most distinctive feature of LEAPs is their strong IDP character (except class 7), which can however be clustered into two different sets. The first set includes classes [1 to 6], with largely unstructured proteins, the second set includes classes [7], [8], with a higher level of structure in native conditions. This is clearly corroborated by their global physico-chemical properties (especially FoldIndex and GRAVY) and amino acid usage. The existence of two types of LEAP could possibly be related to stress intensity. LEAPs with higher structural order would be rapidly functional under moderate stress such as the onset of dehydration, while largely unstructured LEAPs would be mobilized through structural changes induced by severe stress situations. This does not preclude the possibility that some LEAPs would be functional in the intrinsically disordered state. The diversity of LEAP sequences thus fits well with a function as genuine IDPs: indeed they represent a large panel of proteins with flexible conformations thereby allowing a variety of interactions with multiple partners.

## Materials and Methods

Many graphics shown in this study and many others can be automatically generated online using the «Statistical analysis» option of the web interface of LEAPdb (http://forge.info.univ-angers.fr/~gh/Leadb/index.php).

### Consensus sequences of the LEAP classes

These were obtained using Multalin [74]. Alignment of all sequences within each LEAP class was performed with a low consensus value = 35% and a high consensus value = 60% (*i.e.* both above the «twilight zone») with a PAM matrix (since sequences of each LEAP class are either distant or not). Gap penalties values (gap open penalty = 2/gap extension penalty = 0/no gap penalty for extremities) were chosen in order to limit stringent conditions for the alignments, thus introducing numerous gaps (Table 1). This «local - global alignment» of all sequences within each LEAP class leads to a class consensus sequence, revealing a high level of similarity between those sequences, especially in the case of LEAP classes 3, 5, 7, 10, 11 and 12.

### Radial phylogram

Subsequent alignment of the 12 consensus sequences of the LEAP classes was made using ClustalW [75] with Gonnet matrix and the following gap penalties values: gap open = 10/gap extension = 0.2/gap distance = 5/end gap allowed. A distance matrix was calculated either using Neighbour joining or UPGMA, giving the same result. The final radial phylogram was drawn using «Dendroscope» [76].

### Boxplots

Each box encloses 50% of the data with the median value of the variable displayed as a line. The top and bottom of the box mark the limits of ±25% of the variable population. The lines extending from the top and bottom of each box mark the minimum and maximum values within the data set that fall within an acceptable range. Outliers points are those whose values are either greater than upper quartile+(1.5×interquartile distance) or less than lower quartile−(1.5×interquartile distance).

### Mean net charge vs. mean hydrophobicity and mean net charge vs. mean hydropathy plots

The mean net charge at pH 7 is the net charge of the polypeptide at pH 7 calculated using the pKa of the residues divided by the length of the sequence. The mean normalized net charge at pH 7.0 (<R>) is the mean net charge at pH 7.0 normalized between 0 and 1.

GRAVY (grand average of hydropathy) is calculated by adding the hydropathy value of all residues divided by the number of residues in the polypeptide. The hydropathy scale is that of Kyte and Doolittle [34]. The normalized GRAVY is the GRAVY normalized between 0 and 1.

The mean hydrophobicity <H> is the sum of the hydrophobicity, using the hydrophobicity scale of Eisenberg et al. [35], of all residues divided by the number of residues in the polypeptide. The mean normalized hydrophobicity (normalized <H>) is the mean hydrophobicity normalized between 0 and 1.

### Statistical analyses

44 variables were studied: one «class» variable, 12 physico-chemical properties (including the ratio MW/Length), 20 relative percentages of amino acids and 11 combinations of plain percentages of amino acids. Table 2 lists the properties and the combinations studied. We first performed an exhaustive statistical analysis of the 43 variables on all 710 LEAPs and on each of the classes, checking for the normality of the distributions of the variables since some classes have less than 50 proteins. We then performed a non-parametric one-way analysis of variance for each of the 43 quantitative variables with the class variable as factor using the non-parametric *Kruskal-Wallis*' test to determine if there were significant differences between the classes. Then, all 43 quantitative variables were compared with *Spearman*'s correlation coefficient to define groups of highly inter correlated variables. At last, a PCA was used to summarize the main spatial relations between the variables and a coherent hierarchical clustering algorithm was applied (Euclidean distances with *Ward*'s inertia method) on the factorial coordinates in order to evaluate the initial classes. All the statistical analysis and graphics were produced with R software [77].

### LEAP class alignments accessible online (see text S1)

These were made using Multalin with various parameters (matrices and gap penalties). Final alignments and position of motifs characterizing each LEAP class were drawn using ESPript [78].

#### PIDP datasets

A number of sequences corresponding to GRAS proteins (gibberellic acid insensitive (GAI), repressor of GAI, Scarecrow) were collected [79]. Plant IDPs were searched using DisProt [80] and “Entrez” (NCBI). We also searched archetypal IDP or IDR such as p53, abscisic stress ripening protein, CREB-binding protein, proteins related to DNA binding or processing, transcription regulation (cyclin-dependent kinase inhibitor, histone) and specific plants proteins (glutenin, Calvin cycle enzymes). Additional sequences were obtained by BLAST: only sequences having more than 50% identity with the query sequence were kept. Among the results, only fully annotated files corresponding to full-length sequences were retained. Finally, to ensure their IDP character, we retained only sequences with FoldIndex ≤0.

#### FS dataset

A set of fully structured proteins with known 3-D structures was selected from the PDB select 25 file (Feb. 2011 - [81]): all proteins have less than 25% sequence identity with high quality X-ray crystallography resolution (<3.5 Angstroms).

#### Signal and targeting peptide prediction

these were performed for the 12 LEAP classes using TargetP 1.1 [82]. Predictions were confirmed using specific tools for chloroplastic, mitochondrial (MitoProt II - [83]) or secreted (SignalP 4.0 - [84]) proteins in the case of Bacteria or Archea (PSORT - [85]) or Fungi (WoLF PSORT - [86]).

## Supporting Information

Figure S1 **Boxplot representation of isoelectric point, mean net charge at pH 7 and mean hydrophilicity of the 12 LEAP classes, IDP and FS proteins.** The line delimitates the mean value. In the case of isoelectric point, it corresponds to 7. In the case of mean net charge at pH 7 and mean hydrophilicity, it corresponds to 0. (A) Isoelectric point. (B) Mean net charge at pH 7. (C) Mean hydrophilicity.
(TIFF)

Figure S2 **Boxplot representation of mean molar fraction of buried residues, mean molar fraction of accessible residues and mean transmembrane tendency of the 12 LEAP classes, IDP and FS proteins.** The line delimitates the mean or the median value calculated for the 12 LEAP classes. (A) Mean molar fraction of buried residues. (B) Mean transmembrane tendency. (C) Mean molar fraction of accessible residues.
(TIFF)

Figure S3 **Boxplot representation of Grand average of hydropathy (GRAVY) and mean hydrophobicity <H> of the 12 LEAP classes, IDP and FS proteins.** The line delimitates the mean value. In both cases, it corresponds to 0. (A) GRAVY. (B) Mean hydrophobicity <H>.
(TIFF)

Figure S4 **Boxplot representation of Gly, Cys, Asn and Gln usage by the 12 LEAP classes, IDP and FS proteins.** The percentage of each amino acid was first calculated for each LEAP class. This value was then divided by the percentage of each amino acid found in the release 2010_04 of UniProtKB/Swiss-Prot [40]. This ratio thus describes the frequency of usage of each amino acid by LEAPs. The line corresponds to a ratio equal to 1.
(TIFF)

Figure S5 **Boxplot representation of Phe, Tyr, Trp and His usage by the 12 LEAP classes, IDP and FS proteins.** The percentage of each amino acid was first calculated for each LEAP class. This value was then divided by the percentage of each amino acid found in the release 2010_04 of UniProtKB/Swiss-Prot [40]. This ratio thus describes the frequency of usage of each amino acid by LEAPs. The line corresponds to a ratio equal to 1.
(TIFF)

Figure S6 **Boxplot representation of Ala, Leu, Ile and Val usage by the 12 LEAP classes, IDP and FS proteins.** The percentage of each amino acid was first calculated for each LEAP class. This value was then divided by the percentage of each amino acid found in the release 2010_04 of UniProtKB/Swiss-Prot [40]. This ratio thus describes the frequency of usage of each amino acid by LEAPs. The line corresponds to a ratio equal to 1.
(TIFF)

Figure S7 **Boxplot representation of Ser, Thr, Met and Pro usage by the 12 LEAP classes, IDP and FS proteins.** The percentage of each amino acid was first calculated for each LEAP class. This value was then divided by the percentage of each amino acid found in the release 2010_04 of UniProtKB/Swiss-Prot [40]. This ratio thus describes the frequency of usage of each amino acid by LEAPs. The line corresponds to a ratio equal to 1.
(TIFF)

Figure S8 **Fractional content (** ***i.e.*** **the sum of residues normalized by protein chain-length) of some particular amino acids combinations.** (A) Positively charged residues [K+R]. (B) Negatively charged residues [D+E]. (C) Strongest disorder promoting residues [R+E+S+P]. (D) Strongest order promoting residues [C+F+Y+W].
(TIFF)

Figure S9 **The distribution of net charge at pH 7 for all 710 LEAPs (A to C) contained in LEAPdb** [**8**] **and for the 12 LEAP classes (D to O).** (A), (B) and (C) show the global normal distribution of net charge. Graphics (D) to (O) correspond to the distribution of net charge by class, revealing its non-normality among the classes. The red line corresponds to the normal distribution associated to the data and the blue line corresponds to the estimated density curve.
(TIFF)

Figure S10 **The means of the classes as supplementary variables of the PCA and their confidence ellipses.** All proteins are plotted as dots in the main plane of the PCA (axis I and II). Variables are added with their names and classes are represented by the projection of their mean plus the corresponding confidence ellipse.
(TIFF)

Figure S11 **Boxplots showing the difference among the 12 LEAP classes, IDP and FS proteins for the variables [D+E+K+R], [D+E−K−R] and [A+I+L+V].** (A) Combination [D+E+K+R]. (B) Combination [D+E−K−R]. (C) Combination [A+I+L+V].
(TIFF)

Figure S12 **Part of the HCA for the PCA.** Some aggregations of the HCA are shown, at a low hierarchical level. The classes are printed with different colors. No early cluster corresponds exactly to a class.
(TIFF)

Figure S13 **Mean normalized hydrophobicity (<H>)** ***vs.*** **mean net charge (<R>) plots for LEAP classes 1 to 6.** The line indicates the boundary between folded (above) and unfolded (below) polypeptide chains. The figure for LEAP class 1 is the same as that of Figure 6.
(TIFF)

Figure S14 **Mean normalized hydrophobicity (<H>)** ***vs.*** **mean net charge (<R>) plots for LEAP classes 7 to 12.** The line indicates the boundary between folded (above) and unfolded (below) polypeptide chains. The figure for LEAP class 7 is the same as that of Figure 6.
(TIFF)

Text S1 **Alignments of LEAPs accessible online.** Only parts of sequences around the motifs are presented in the figures. Amino acids of the motifs are indicated at the bottom of alignments.
(DOC)
