# Supplementary figures and images for: Computational and Statistical Analyses of Amino Acid Usage and Physico-Chemical Properties of the Twelve Late Embryogenesis Abundant Protein Classes
Source: PLoS One. 2012 May 16;7(5):e36968. doi: 10.1371/journal.pone.0036968 (PMC3353982; doi:10.1371/journal.pone.0036968)

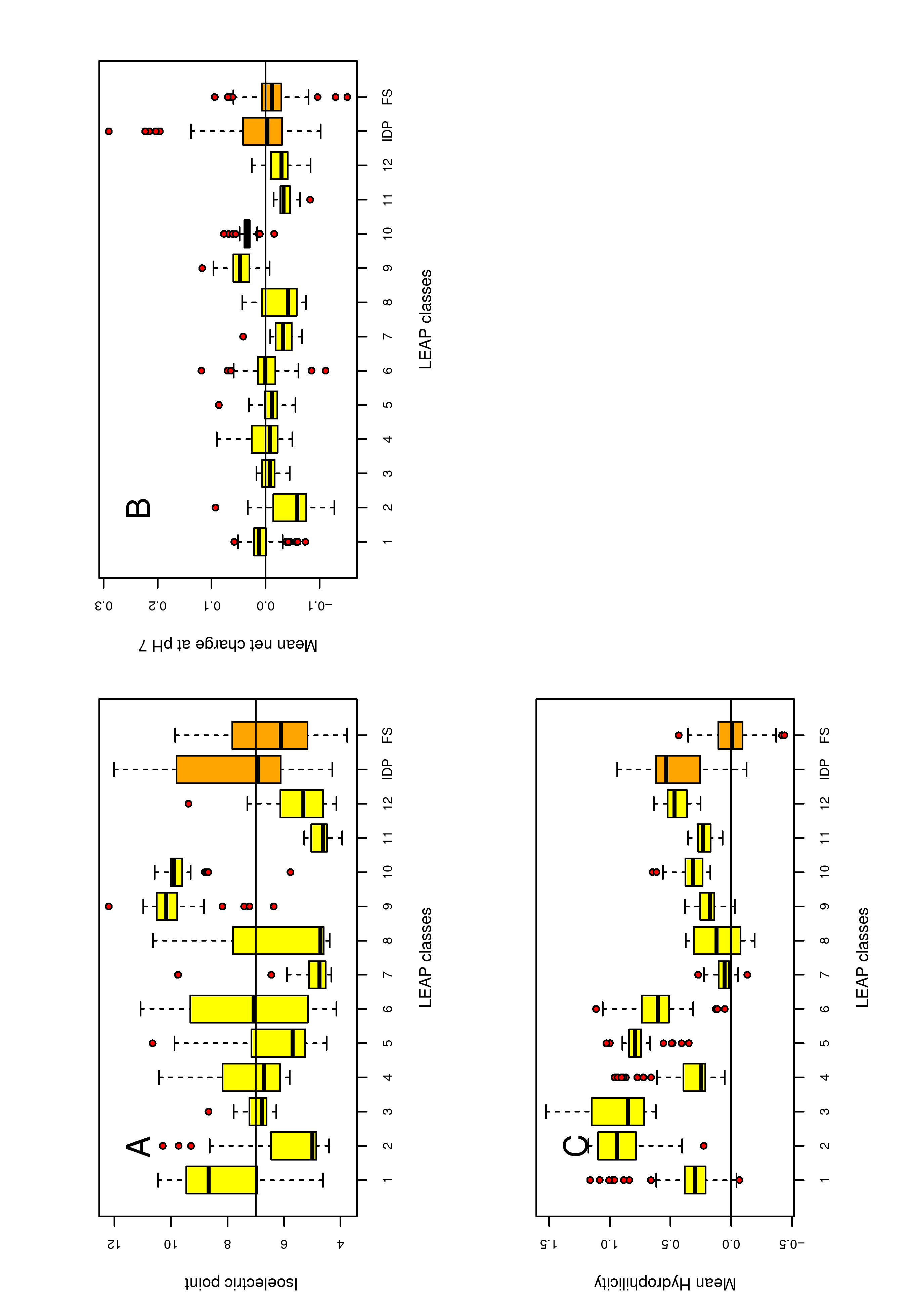

Supplement: Figure S1 — Boxplot representation of isoelectric point, mean net charge at pH 7 and mean hydrophilicity of the 12 LEAP classes, IDP and FS proteins. The line delimitates the mean value. In the case of isoelectric point, it corresponds to 7. In the case of mean net charge at pH 7 and mean hydrophilicity, it corresponds to 0. (A) Isoelectric point. (B) Mean net charge at pH 7. (C) Mean hydrophilicity. (TIFF) [file pone.0036968.s001.tiff]

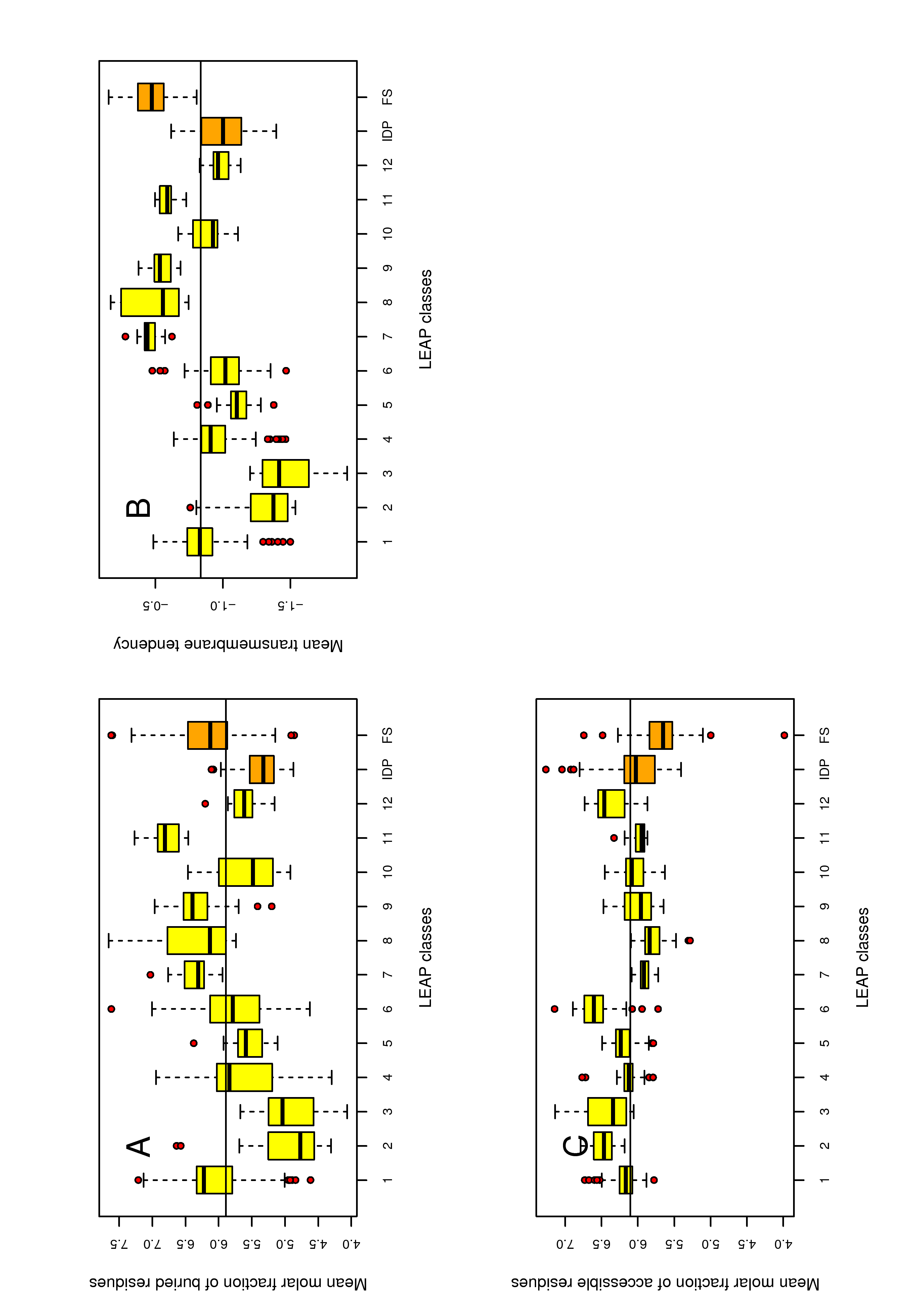

Supplement: Figure S2 — Boxplot representation of mean molar fraction of buried residues, mean molar fraction of accessible residues and mean transmembrane tendency of the 12 LEAP classes, IDP and FS proteins. The line delimitates the mean or the median value calculated for the 12 LEAP classes. (A) Mean molar fraction of buried residues. (B) Mean transmembrane tendency. (C) Mean molar fraction of accessible residues. (TIFF) [file pone.0036968.s002.tiff]

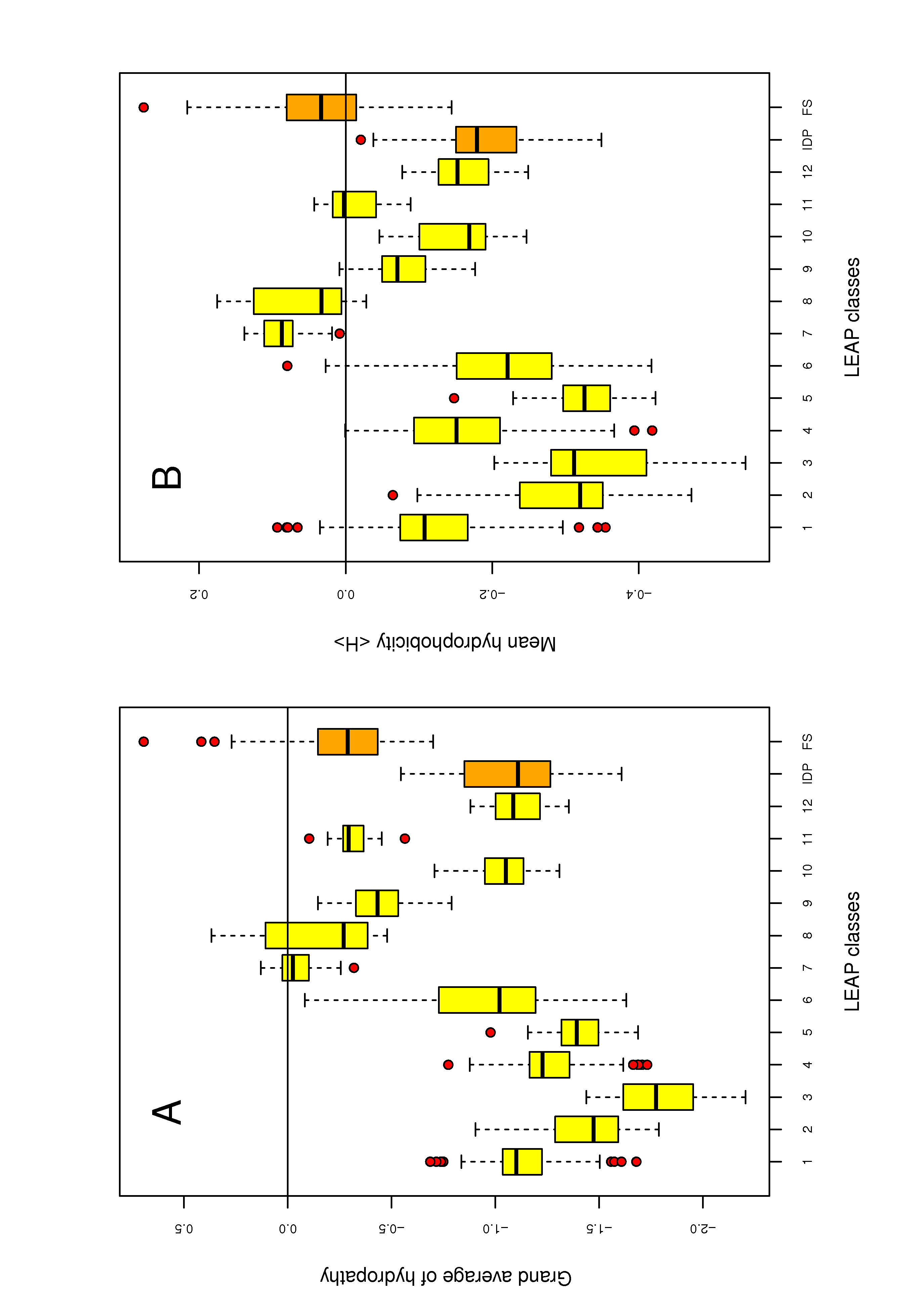

Supplement: Figure S3 — Boxplot representation of Grand average of hydropathy (GRAVY) and mean hydrophobicity of the 12 LEAP classes, IDP and FS proteins. The line delimitates the mean value. In both cases, it corresponds to 0. (A) GRAVY. (B) Mean hydrophobicity . (TIFF) [file pone.0036968.s003.tiff]

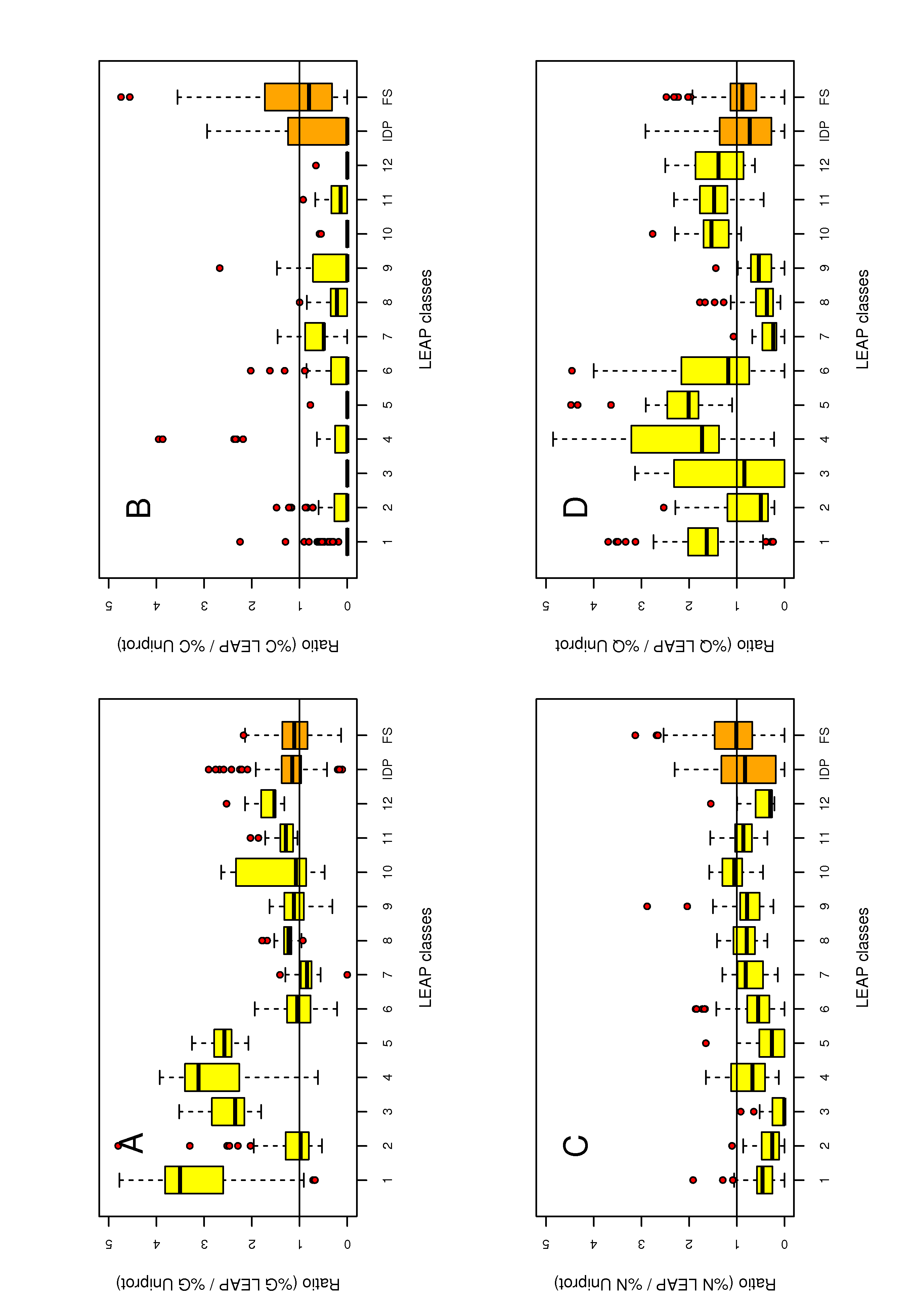

Supplement: Figure S4 — Boxplot representation of Gly, Cys, Asn and Gln usage by the 12 LEAP classes, IDP and FS proteins. The percentage of each amino acid was first calculated for each LEAP class. This value was then divided by the percentage of each amino acid found in the release 2010_04 of UniProtKB/Swiss-Prot [40]. This ratio thus describes the frequency of usage of each amino acid by LEAPs. The line corresponds to a ratio equal to 1. (TIFF) [file pone.0036968.s004.tiff]

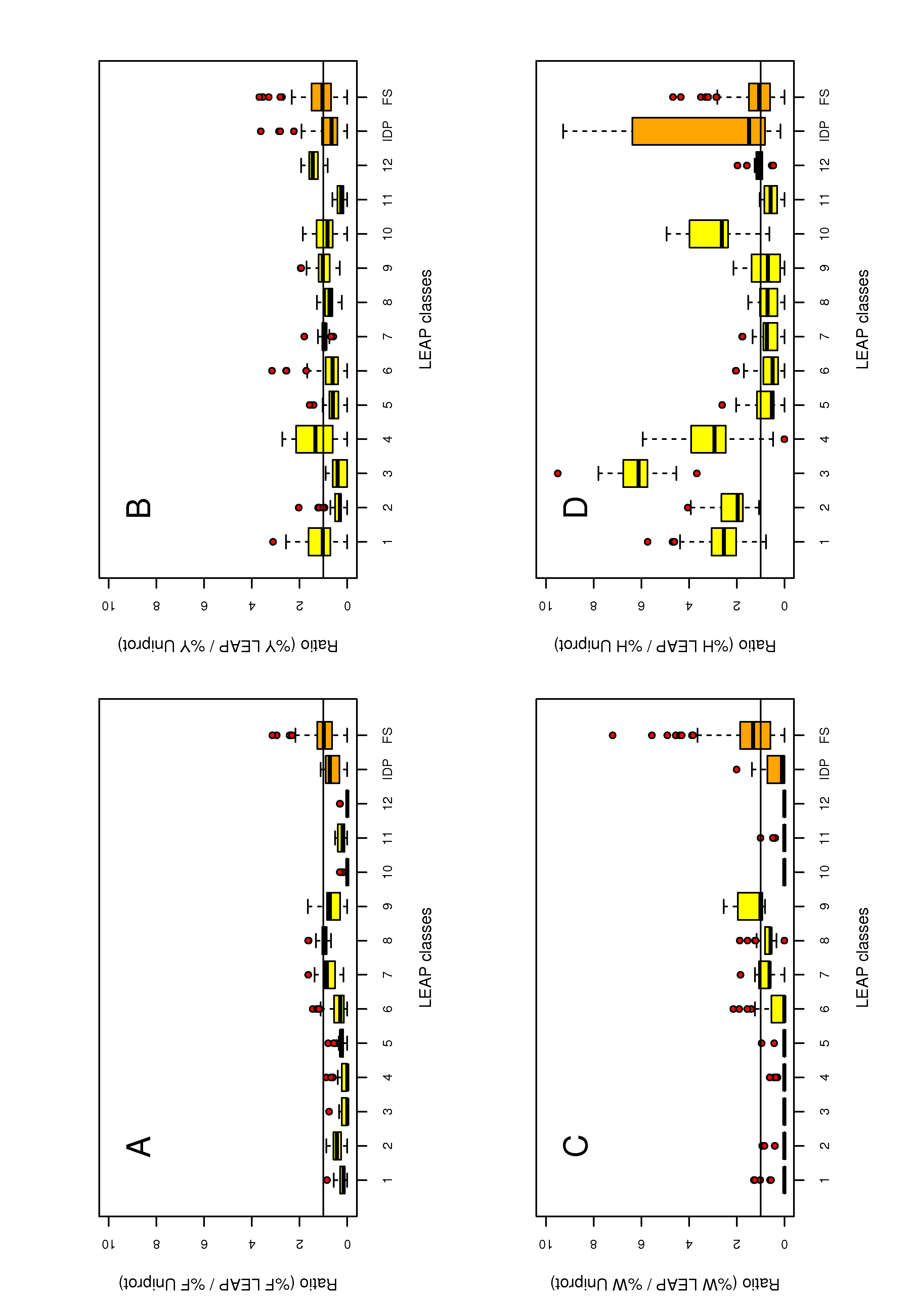

Supplement: Figure S5 — Boxplot representation of Phe, Tyr, Trp and His usage by the 12 LEAP classes, IDP and FS proteins. The percentage of each amino acid was first calculated for each LEAP class. This value was then divided by the percentage of each amino acid found in the release 2010_04 of UniProtKB/Swiss-Prot [40]. This ratio thus describes the frequency of usage of each amino acid by LEAPs. The line corresponds to a ratio equal to 1. (TIFF) [file pone.0036968.s005.tiff]

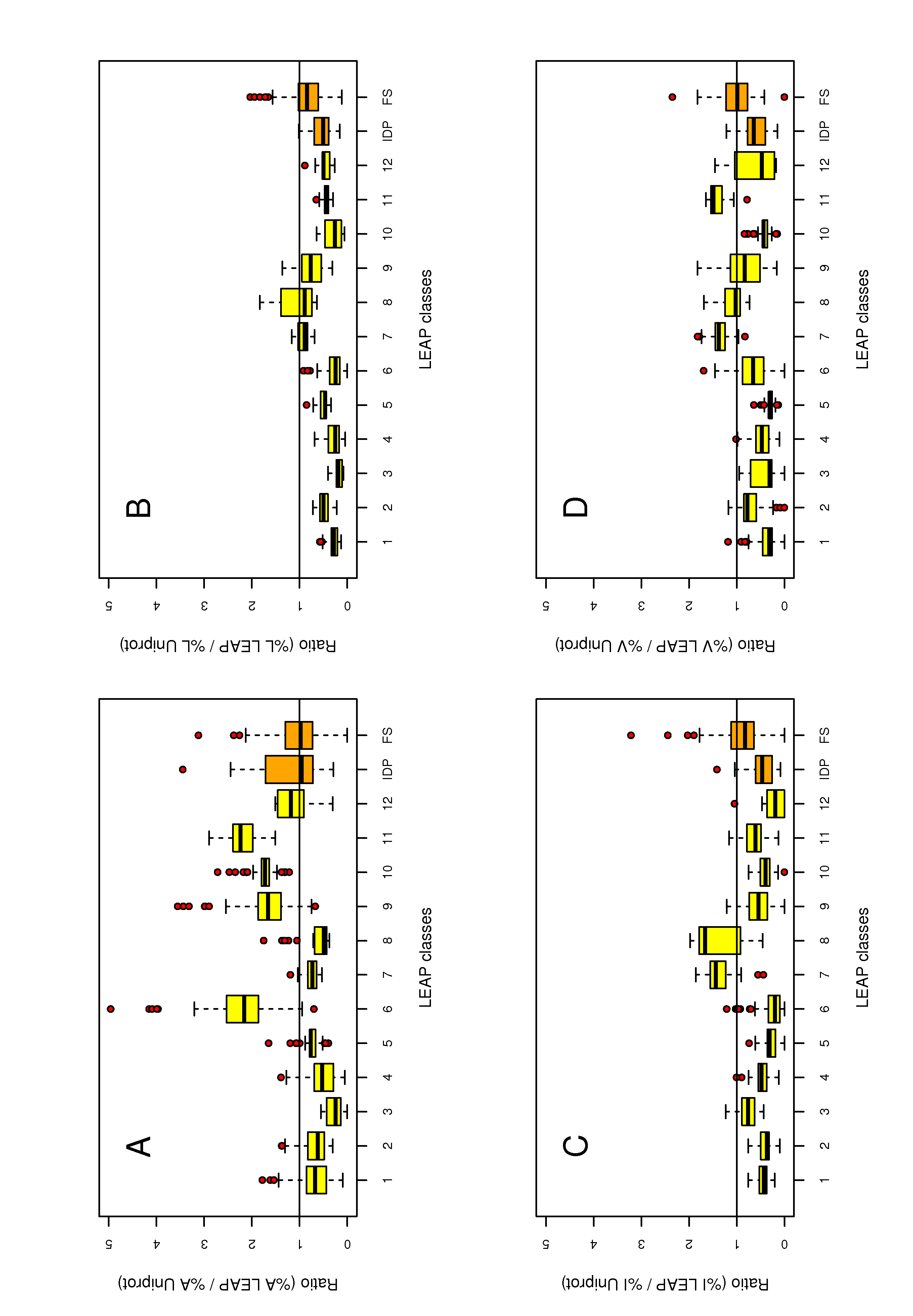

Supplement: Figure S6 — Boxplot representation of Ala, Leu, Ile and Val usage by the 12 LEAP classes, IDP and FS proteins. The percentage of each amino acid was first calculated for each LEAP class. This value was then divided by the percentage of each amino acid found in the release 2010_04 of UniProtKB/Swiss-Prot [40]. This ratio thus describes the frequency of usage of each amino acid by LEAPs. The line corresponds to a ratio equal to 1. (TIFF) [file pone.0036968.s006.tiff]

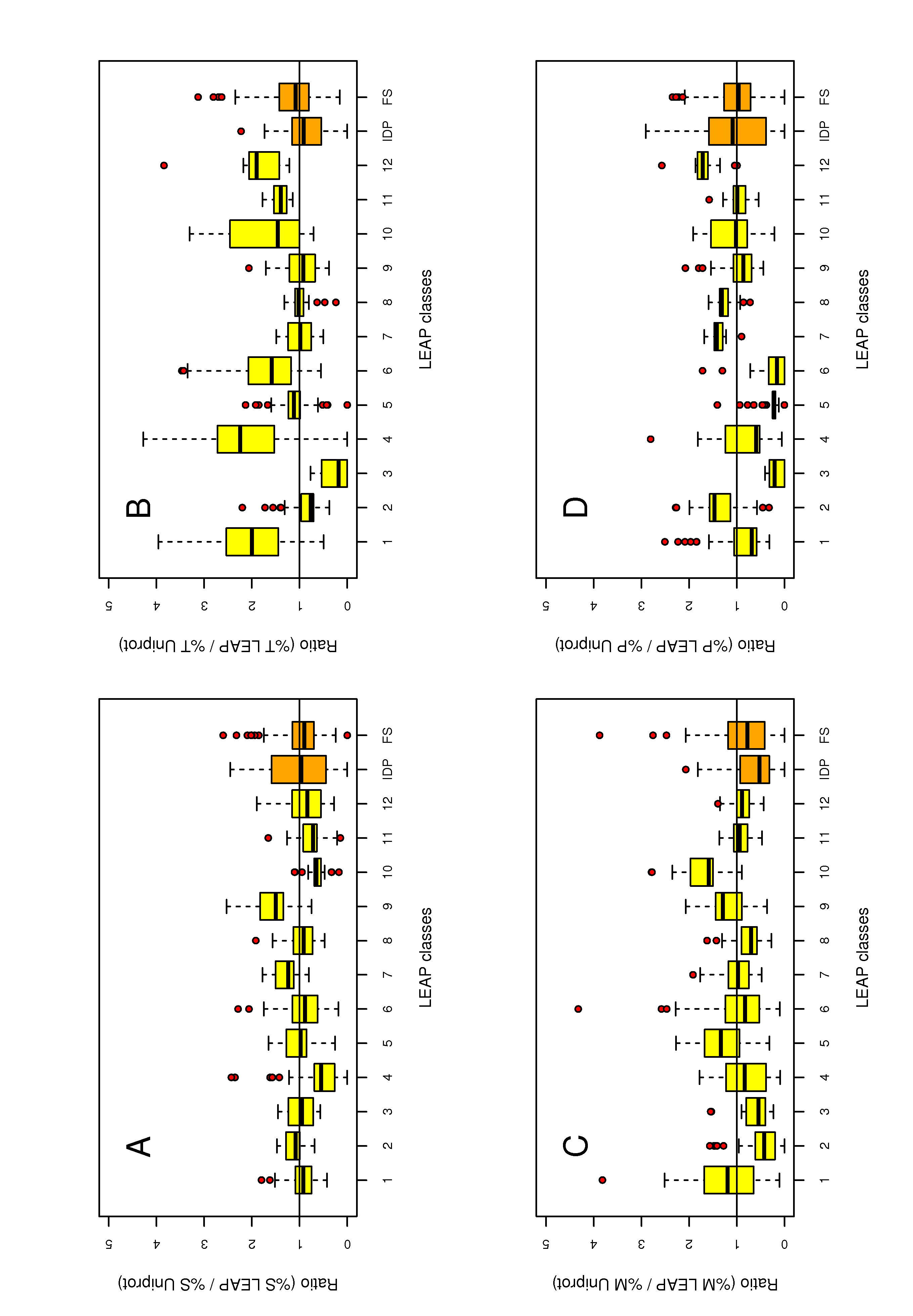

Supplement: Figure S7 — Boxplot representation of Ser, Thr, Met and Pro usage by the 12 LEAP classes, IDP and FS proteins. The percentage of each amino acid was first calculated for each LEAP class. This value was then divided by the percentage of each amino acid found in the release 2010_04 of UniProtKB/Swiss-Prot [40]. This ratio thus describes the frequency of usage of each amino acid by LEAPs. The line corresponds to a ratio equal to 1. (TIFF) [file pone.0036968.s007.tiff]

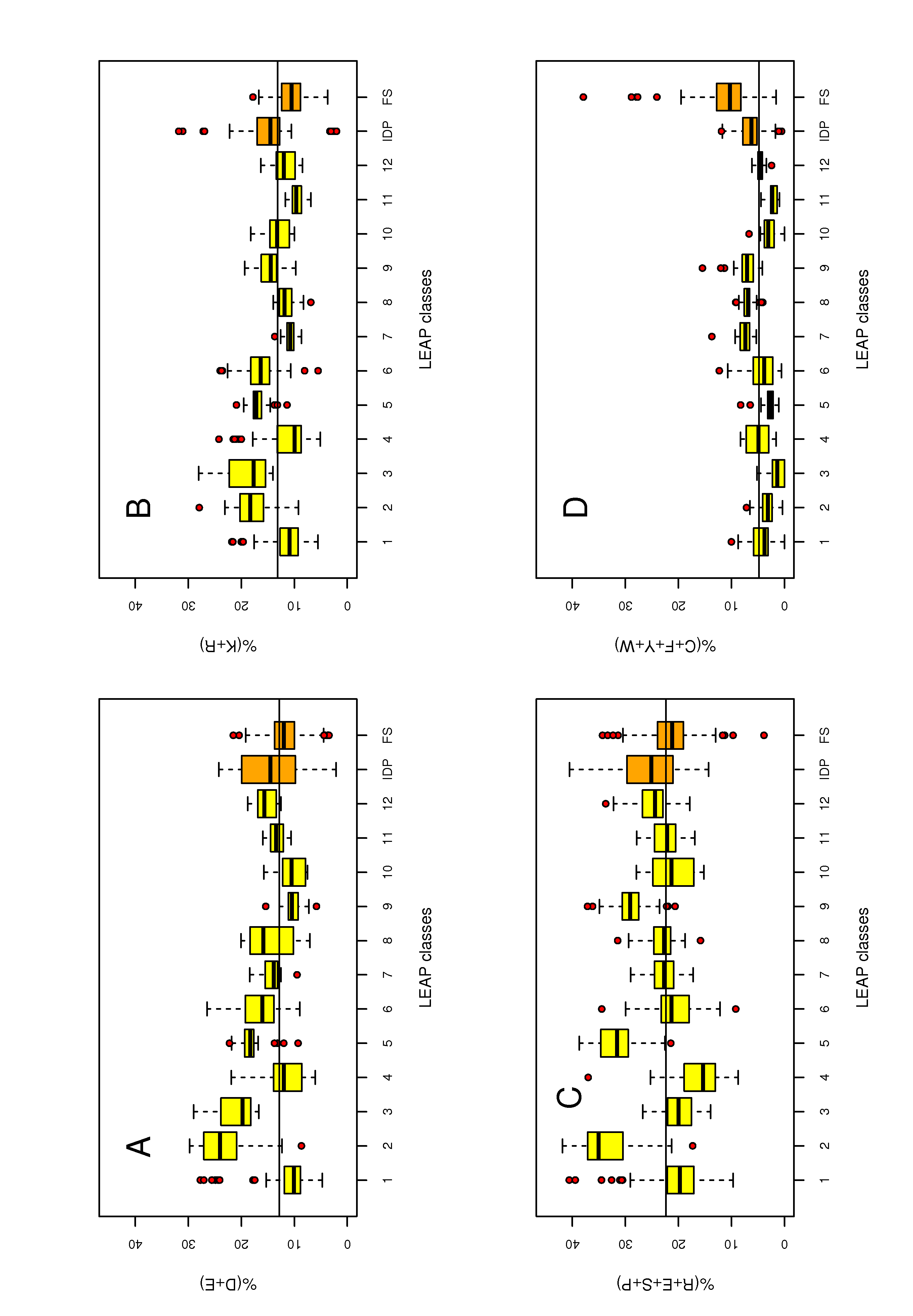

Supplement: Figure S8 — Fractional content ( i.e. the sum of residues normalized by protein chain-length) of some particular amino acids combinations. (A) Positively charged residues [K+R]. (B) Negatively charged residues [D+E]. (C) Strongest disorder promoting residues [R+E+S+P]. (D) Strongest order promoting residues [C+F+Y+W]. (TIFF) [file pone.0036968.s008.tiff]

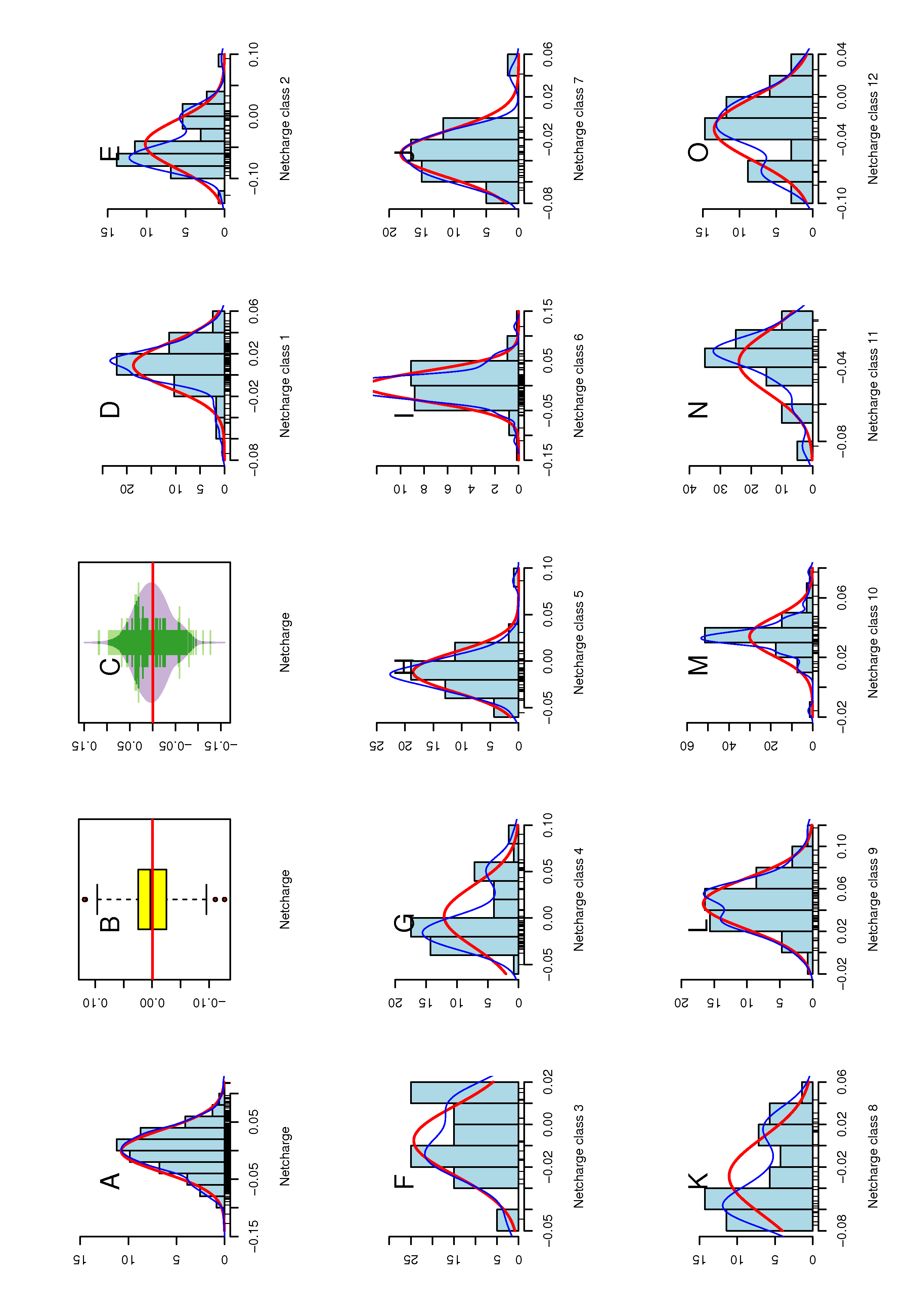

Supplement: Figure S9 — The distribution of net charge at pH 7 for all 710 LEAPs (A to C) contained in LEAPdb [8] and for the 12 LEAP classes (D to O). (A), (B) and (C) show the global normal distribution of net charge. Graphics (D) to (O) correspond to the distribution of net charge by class, revealing its non-normality among the classes. The red line corresponds to the normal distribution associated to the data and the blue line corresponds to the estimated density curve. (TIFF) [file pone.0036968.s009.tiff]

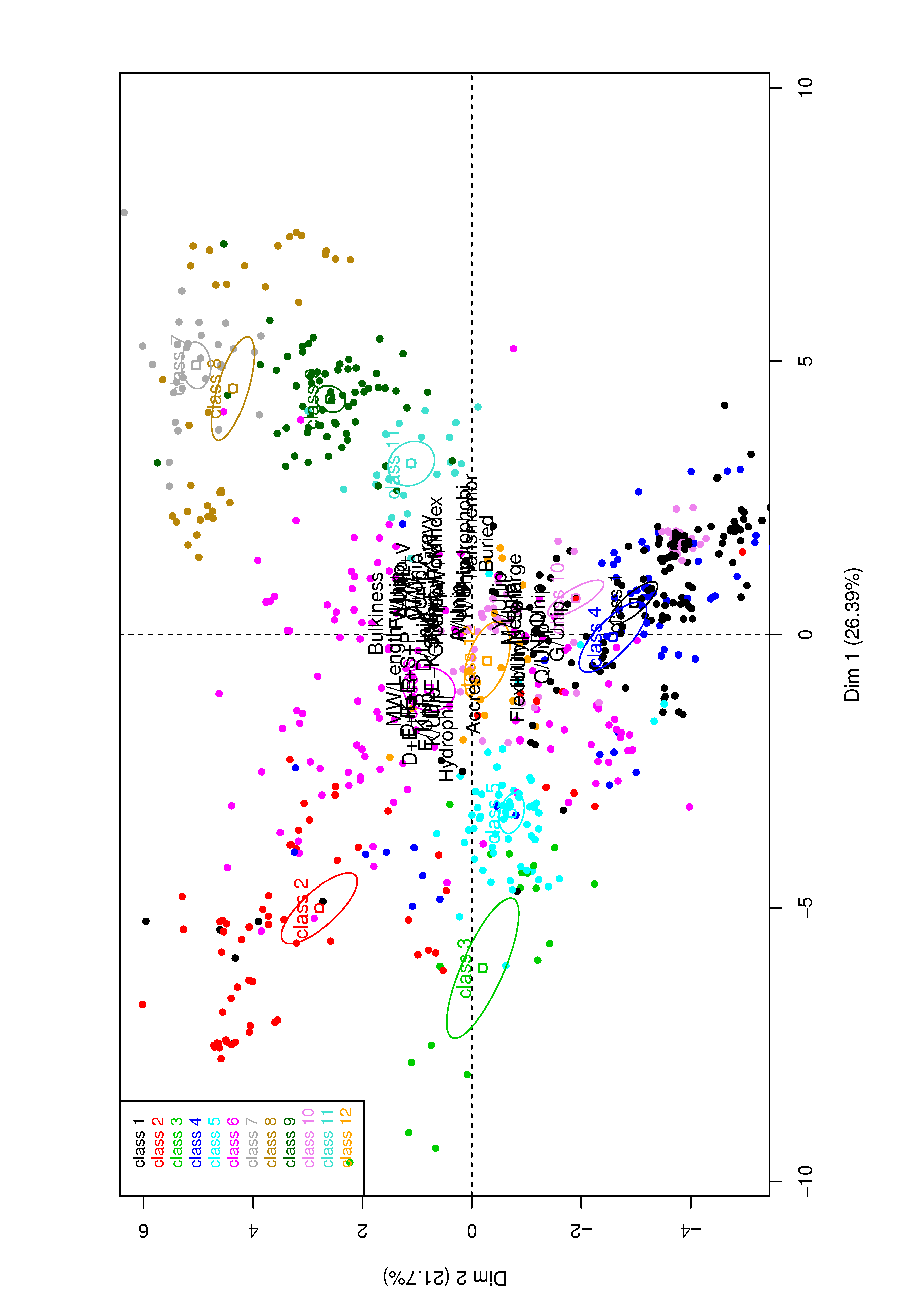

Supplement: Figure S10 — The means of the classes as supplementary variables of the PCA and their confidence ellipses. All proteins are plotted as dots in the main plane of the PCA (axis I and II). Variables are added with their names and classes are represented by the projection of their mean plus the corresponding confidence ellipse. (TIFF) [file pone.0036968.s010.tiff]

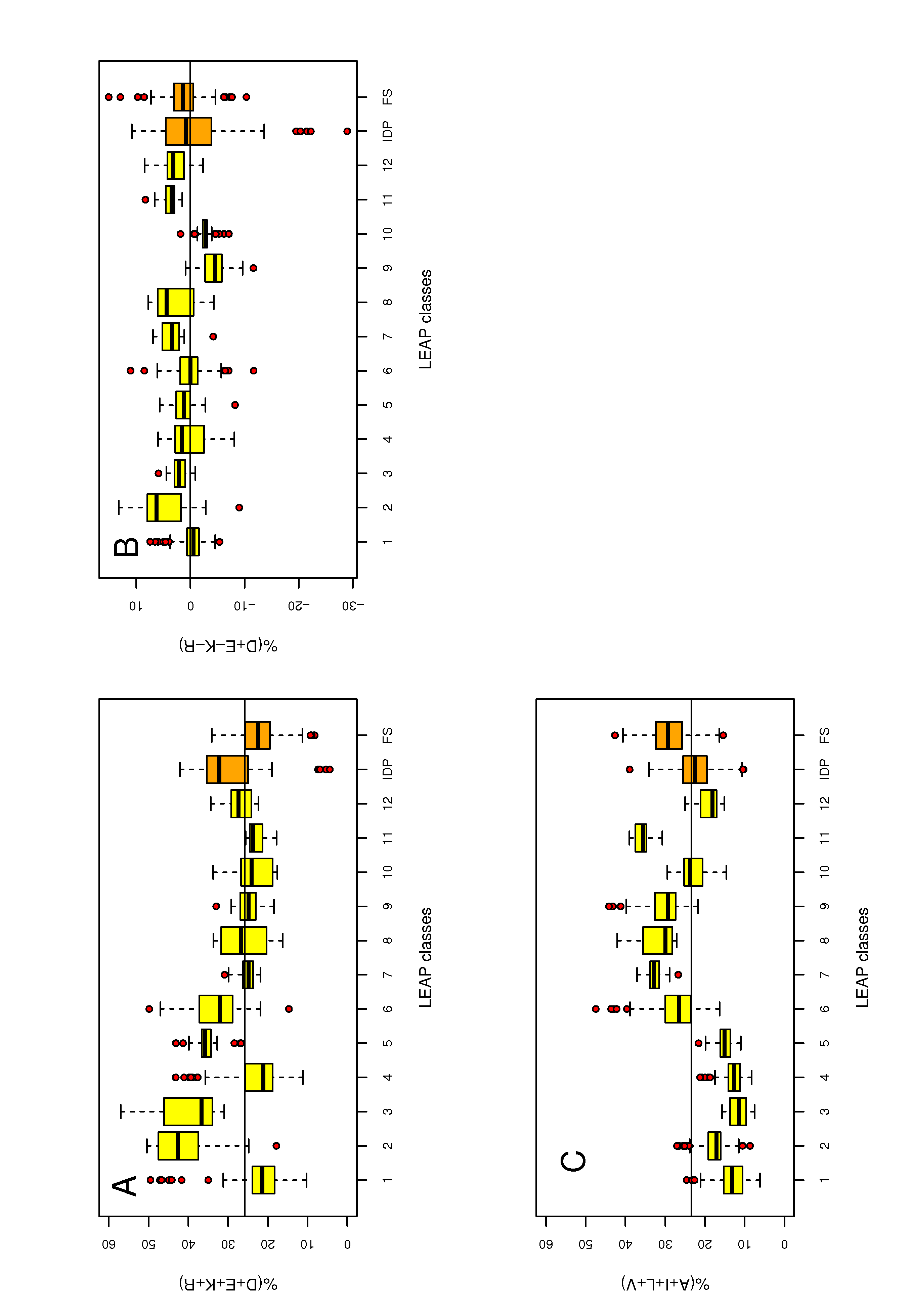

Supplement: Figure S11 — Boxplots showing the difference among the 12 LEAP classes, IDP and FS proteins for the variables [D+E+K+R], [D+E−K−R] and [A+I+L+V]. (A) Combination [D+E+K+R]. (B) Combination [D+E−K−R]. (C) Combination [A+I+L+V]. (TIFF) [file pone.0036968.s011.tiff]

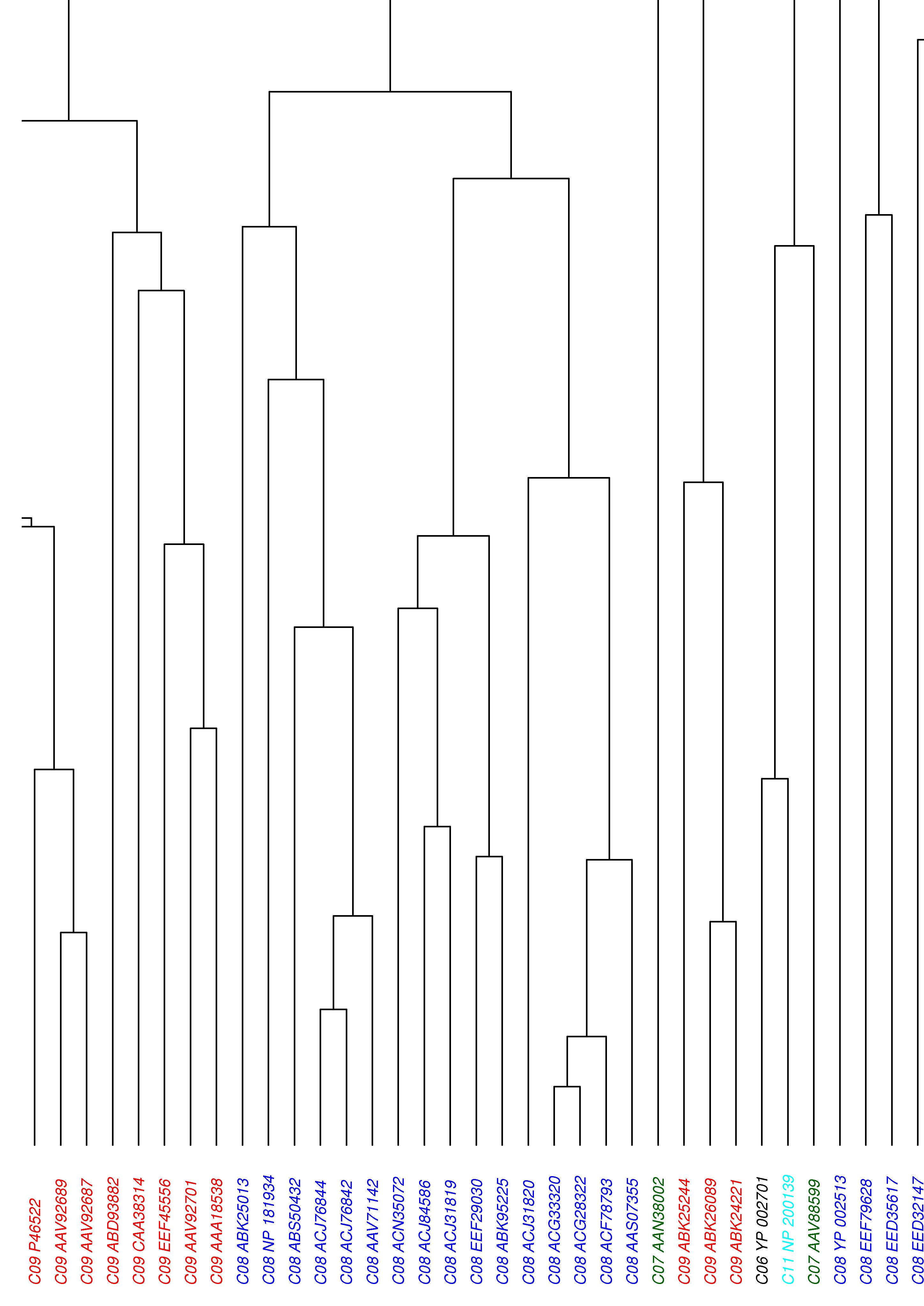

Supplement: Figure S12 — Part of the HCA for the PCA. Some aggregations of the HCA are shown, at a low hierarchical level. The classes are printed with different colors. No early cluster corresponds exactly to a class. (TIFF) [file pone.0036968.s012.tiff]

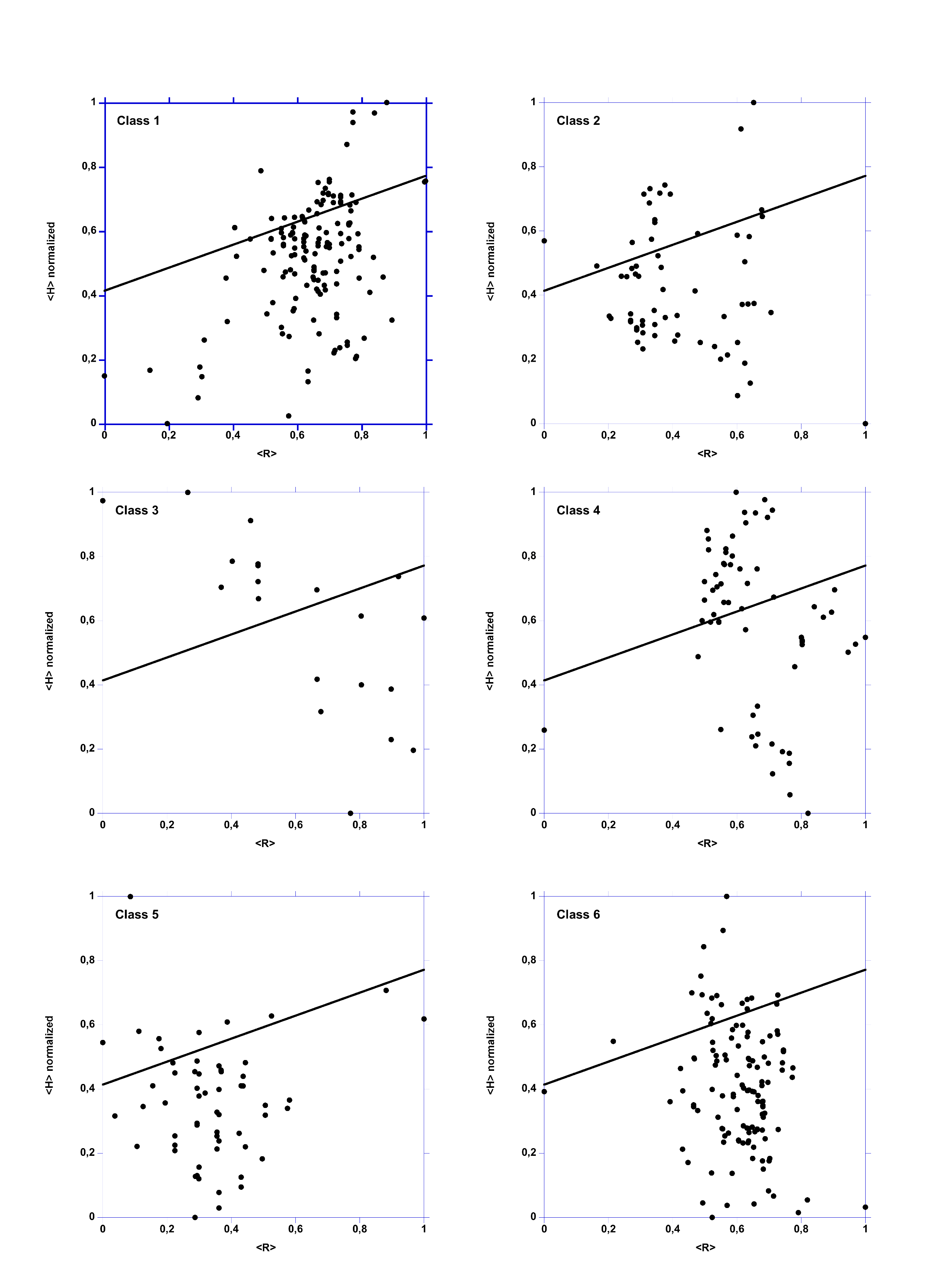

Supplement: Figure S13 — Mean normalized hydrophobicity () vs. mean net charge () plots for LEAP classes 1 to 6. The line indicates the boundary between folded (above) and unfolded (below) polypeptide chains. The figure for LEAP class 1 is the same as that of Figure 6. (TIFF) [file pone.0036968.s013.tiff]

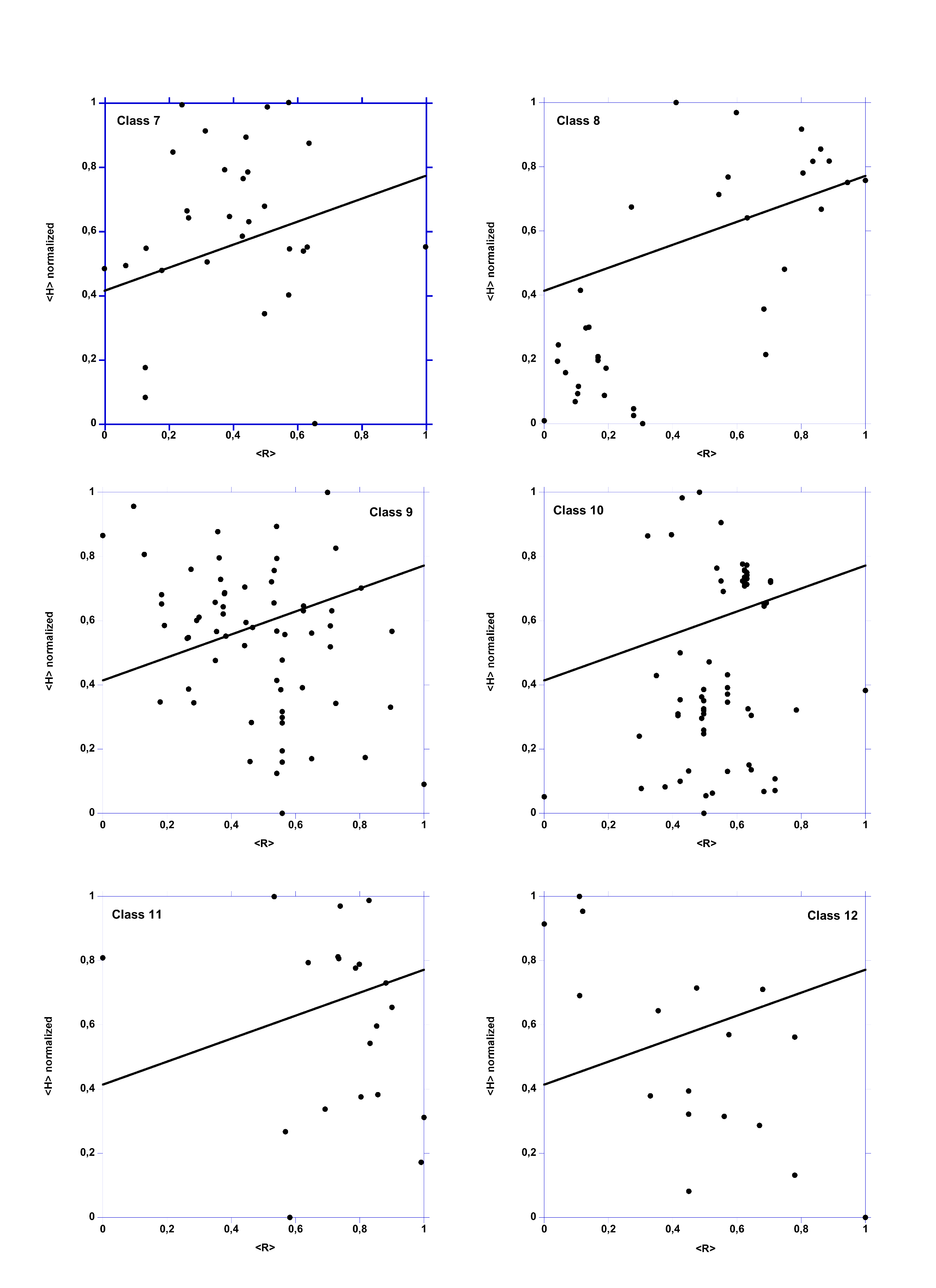

Supplement: Figure S14 — Mean normalized hydrophobicity () vs. mean net charge () plots for LEAP classes 7 to 12. The line indicates the boundary between folded (above) and unfolded (below) polypeptide chains. The figure for LEAP class 7 is the same as that of Figure 6. (TIFF) [file pone.0036968.s014.tiff]
