# Supplementary material for: Computational and Statistical Analyses of Amino Acid Usage and Physico-Chemical Properties of the Twelve Late Embryogenesis Abundant Protein Classes
Source: PLoS One. 2012 May 16;7(5):e36968. doi: 10.1371/journal.pone.0036968 (PMC3353982; doi:10.1371/journal.pone.0036968)
Supplement: Text S1 — Alignments of LEAPs accessible online. Only parts of sequences around the motifs are presented in the figures. Amino acids of the motifs are indicated at the bottom of alignments. (DOC) [file pone.0036968.s015.doc]

# SUPPORTING INFORMATION

## Text S1. Alignments of LEAPs accessible online at <http://forge.info.univ-angers.fr/~gh/Leadb/index.php?action=2&mode=8>.

Only parts of sequences around the motifs are presented in the figures. Amino acids of the motifs are indicated at the bottom of alignments.

*Class 1:* since the number of sequences to be aligned was very large (145), only 69 sequences are presented in the figure.

*Class 4:* this class belongs to PF00257 and shares the motif « KIKEKLPG » with classes 1, 2 and 3. However this motif does not discriminate these four classes. Therefore, motifs for class 4 are somewhat more complexes and variables (supporting information - Table S1). Most of them are found in the C-terminal of the polypeptide chain as indicated by the box in the figure.

*Class 6:* since the number of sequences to be aligned was very large (125), only 41 sequences are presented in the figure. Although this class is the less conserved, a motif has been found (supporting information - Table S1): it surrounds a first couple of key residues « [KR][AT] » (indicated by plain circles in the figure) and a second couple of key residues « K[AD] ». This second motif is situated 1 to 67 residues forward and is thus indicated in the figure by various positions of stars.

*Class 7:* PDB code 1XO8 (Acc. # O03983 in LEAPdb) was used as template for secondary structures.

*Class 9:* although W is largely under-represented among LEAP classes, the motif of this class contains a conserved W.

*Class 11:* the two possible motifs are shown (plain circles or stars).
